# Solubility model of metal complex in ionic liquids from first principle calculations†

**DOI:** 10.1039/c9ra04042k

**Published:** 2019-06-12

**Authors:** Anwesa Karmakar, Rangachary Mukundan, Ping Yang, Enrique R. Batista

**Affiliations:** Theoretical Division, Los Alamos National Laboratory Los Alamos 87545 USA anwesak@lanl.gov anwesa.karmakar@gmail.com pyang@lanl.gov erb@lanl.gov; MPA-11 Division, Los Alamos National Laboratory Los Alamos 87545 USA

## Abstract

A predictive model based on first principles calculations has been proposed to study the solid–liquid equilibria comprising of metal complexes and ionic liquids. The model is based on first principle COSMO calculation followed by post statistical thermodynamical treatment of self-consistent properties of solute and solvent molecules. The metal complex and ionic liquid have been treated as a simple binary mixture. The ionic liquid has been treated here as a single intact molecule. The experimentally observed dual-solute relationship between the ionic liquid and redox active species in presence of a third organic solvent has been established using our model in this work. Within the model, the dual-solute relationship appeared as a simple Gibbs–Duhem relationship between these two species at ambient condition. The dual-solute relationship between the metal complex (V(acac)_3_, Cr(acac)_3_ and Mn(acac)_3_) and ionic liquid ([Tea][BF_4_]) has been validated by calculating the Gibbs–Duhem relationship, *x*_solute_*vs. x*_solvent(IL)_ and 1/*γ*_solute_*vs. x*_solvent(IL)_ plots. The present model has been applied to a set of ionic liquids, metal complexes and organic solvent (acetonitrile) for which experimental study has been done. The solvation mechanism of the metal complexes in those ionic liquids was obtained using the model. According to our findings, the ionic liquid containing imidazolium cation and [NTf_2_]^−^ anion is appeared as a suitable solvent for the non-aqueous redox flow cell. We have compared our results with the already reported experimental results where they were available for the non-aqueous solvents.

## Abstract 2

A predictive model based on first principles calculations has been proposed to study the solid–liquid equilibria comprising of metal complexes and ionic liquids.

## Graphical abstract

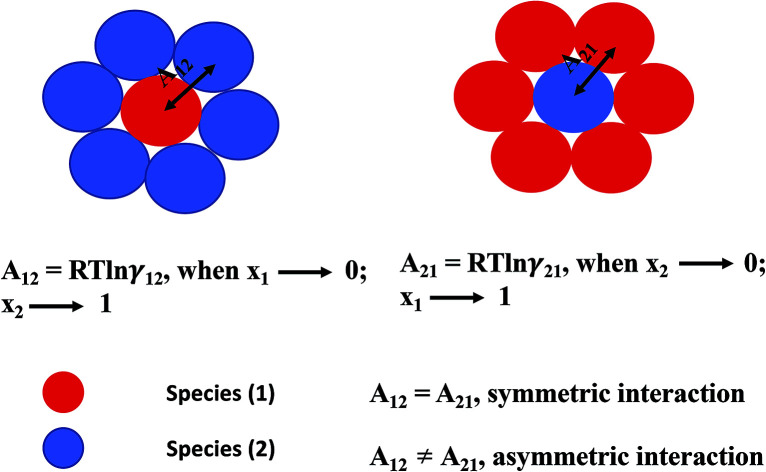

## 1 Introduction

Ionic liquids have emerged as potential non-aqueous media in redox flow batteries.^1–21^ A key property affecting the performance of a flow cell is the solubility of the redox active material in the supporting electrolytes, therefore, a good solubility model is essential to predict the performance of these systems. Solubility has been modeled in several ways using Hansen solubility parameters, Scatchard–Hildebrand solubility,^22^ and first principle-based COSMO-RS^23–25^ calculations followed by statistical mechanical treatment of the self-consistent properties obtained from the quantum calculations for solid dissolution in liquid. However the first two empirical models depend on parameters to be extracted from experimental data and they depend on the nature of the solvating media. Therefore, if experimental results are not available for a specific solvating media, the solubility of that material cannot be predicted. Also, to predict the solubility of solid in liquids, one needs to have apriori knowledge of heat of fusion and melting temperature of those material, which has limited the use of COSMO^24,25^ based solubility models. In this work we show how one can predict a good trend of solubility of the redox active materials in ionic liquids without having any apriori knowledge of heat of fusion and melting temperature using the first-principle COSMOSAC model.^26,27^ These calculations are based on first-principle approach *via* a dielectric continuum model and thus have fewer adjustable parameters than the previous models that can be easily adaptable to the metal complex and ionic liquids. These calculations are fast and with good accuracy. Also the solubility measurement of metal complexes in ionic liquids is a challenging task to do experimentally due to the slow diffusivity of the redox active species through ionic liquids in certain cases.^12,16,18^ Therefore, the model can be used as a very efficient predictive tool for solubility calculations of metal complex in ionic liquids, to screen ionic liquids for the best solubility of certain metal complexes and to study the solvation mechanism of the metal complexes in the ionic liquid and organic solvent.

In a non-aqueous redox flow cell, the redox active species and supporting electrolyte such as ionic liquids are dissolved in the non-aqueous organic solvent medium. The solubility of the redox active species is affected by the presence of the second solute in the medium because its presence changes the activity of the solution. The presence of this second solute may also affect the mobilities of charged species and hence, ionic conductivity of the solution. According to previous studies,^1,9^ the solubility of redox active species decreases with increasing concentration of the supporting electrolyte (IL) which serves as the second solute and the acetonitrile (ACN) as solvent. This phenomenon is known as the dual-solute effect in solubility.^1,9^ However, establishing such relationship experimentally is very difficult. Such relationship can be studied directly by measuring the interaction between the redox active species and the ionic liquids in terms of the non-ideality correction (activity coefficient) at infinite dilution (*γ*^∞^). In this paper, we repeat calculations of vanadium tris acetylacetonato (V(acac)_3_), chromium tris acetylacetonato (Cr(acac)_3_) and manganese tris acetylacetonato (Mn(acac)_3_) in non-aqueous medium such as ionic liquids and organic solvent.

In Section 2, the details of solubility theory has been discussed. The computational details have been discussed in Section 3. The discussion on metal complex σ profile and on ionic liquids σ profile have been given in Subsection 4.1 and 4.2, respectively. The dependence of σ profile on the metal complex conformation has been given in Subsection 4.3. The calculations of solubility, screening of ionic liquid and tests of sensitivity with respect to the model parameters have been discussed in Section 5. The theoretical modeling of dual-solute effect has been explained in Section 6. A brief conclusion is given in Section 7.

## 2 Theory

The solubility of the metal complexes was calculated using a new model here referred as COSMOSAC-LANL activity coefficient model. This model includes two terms: A 3-suffix Margules (3sM)^28,29^ function as a quantitative measurement of inherent asymmetric interaction present in a solution due to the hydrogen bond interactions, strong dispersion, short-range entropic contribution and coulombic interactions and the long-range Staverman–Guggenheim^30,31^ (SG) combinatorial interactions due to the size and shape differences between a solute and solvent molecules. We will work in the regime where *γ*^LANL^_i/S_ ≥ 1 *i.e.*; ln *γ*^LANL^_i/S_ ≥ 0 since we are dealing with the solid–liquid equilibria at low pressure. Hence, we invoke a new asymmetric interaction in terms of “LANL” activity coefficient model

1 ln(*γ*^LANL^_i/S_) = ln(*γ*^comb^_i/S_) + ln(*γ*^asym^_i/S_),

where, we define ln(*γ*^asym^_i/S_) = (Δ*G*^asym^_i/S_ − Δ*G*^asym^_i/i_)/*RT* which is the difference between the asymmetric interactions in mixture (i/S) and pure state (i/i), which represents the solvation free energy change in terms of solute and solvent interactions when a solute particle goes into a fixed position in solution from a fixed position in its ideal state. Since “LANL” activity coefficient model has the asymmetric interaction (ln(*γ*^asym^_i/S_)), the total activity coefficient (ln(*γ*^LANL^_i/S_)) model will be also called an asymmetric model. For pure species, the asymmetric interaction is zero, Δ*G*^asym^_i/i_ = 0, and hence, ln(*γ*^asym^_i/S_) = (Δ*G*^asym^_i/S_)/*RT* = (Δ*G*^solv^_i/S_ − Δ*G*^solv^_i/i_)/*RT*. In COSMOSPACE,^32^ the activity coefficient due to the asymmetric interaction is equal to the activity coefficient due to the short range residual interaction (*γ*_residual_) at infinite dilution (*x*_solute=i_ → 0, *x*_solvent=j_ → 1). In COSMOSPACE, the activity coefficient can be written in terms of the segment interaction as2
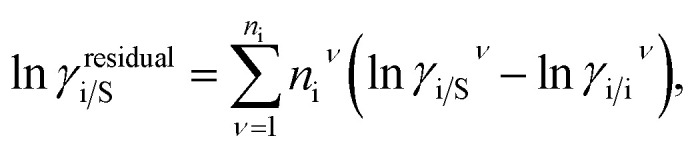
where i stands for the pure compound, *ν* stands for the segment *n*_i_, and *n*_i_ stands for the number of segments and say, (ln *γ*_i/S_^*ν*^ − ln *γ*_i/i_^*ν*^) = ln *γ*_i/S_^*ν*^(asym). The asymmetric interaction mentioned above can be written in terms of asymmetric segment interaction3
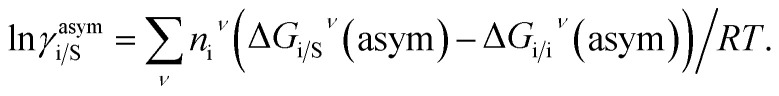
where, Δ*G*_i/i_^*ν*^(asym)/*RT* = 0 for ideal case, so 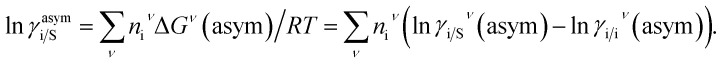 Since for similar type of segments the concept of asymmetric interaction between segments does not exist, so it will vanish and therefore the equation will be reduced to 
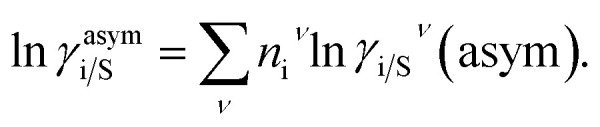
 In this model, we used the 3-suffix Margules function to capture the asymmetric interaction in terms of Margules parameters. Therefore, the expression of the excess Gibbs free energy due to the asymmetric interaction can be written as

**Fig. 1:**
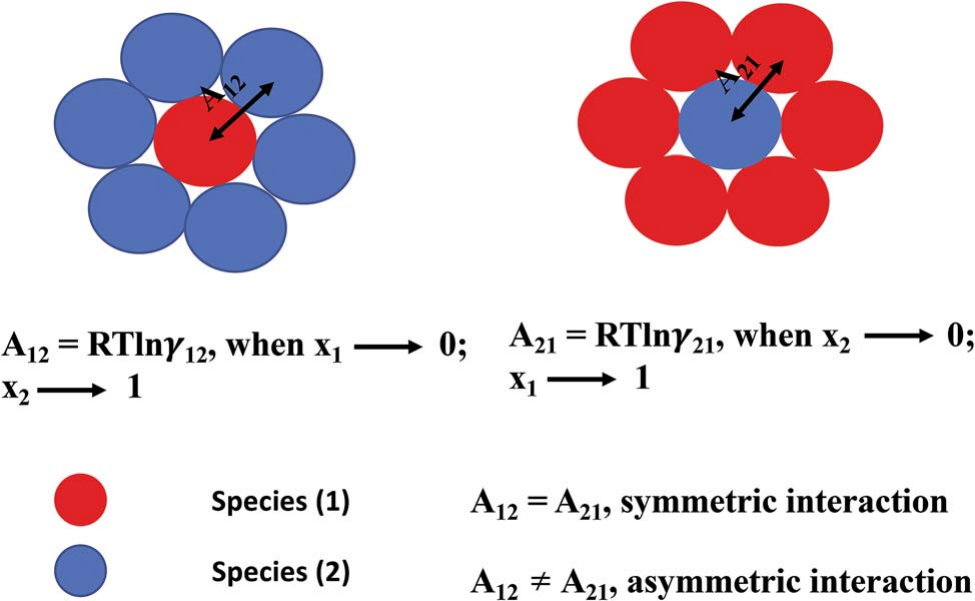
A cartoon representation of asymmetric interaction in a binary mixture at infinite dilution.

4 *G*^ex^ = Δ*G*^asym^_i/S_(real) − Δ*G*^asym^_i/i_(ideal) = *x*_1_*x*_2_(*A*_21_*x*_1_ + *A*_12_*x*_2_),

where, *x*_1_ and *x*_2_ are the mole fractions for solute and solvent molecules in a binary mixture and *A*_21_ and *A*_12_ are the Margules parameters. The solution is asymmetric when *A*_12_ ≠ *A*_21_ and when they are equal (*A*_12_ = *A*_21_), the solution is symmetric reducing to the 2-suffix Margules function. A pictorial representation about obtaining these Margules parameters has been given in Fig. 1. By differentiating eqn (4) with respect to the mole number for species 1 and 2,^33,34^ one can get the asymmetric activity coefficient5
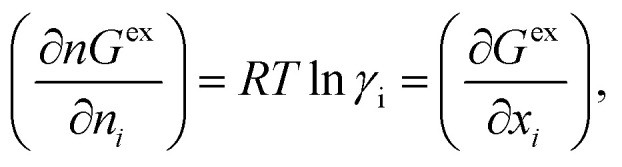
where *n* is the total mole number and *n*_*i*_ is the mole number of species (*i* = 1, 2) and *x*_*i*_ = mole fraction = *n*_*i*_/*n* of *i*th species. One can then get the expression for activity coefficients for both species 1 and 2 present in the binary mixture solution and they are6
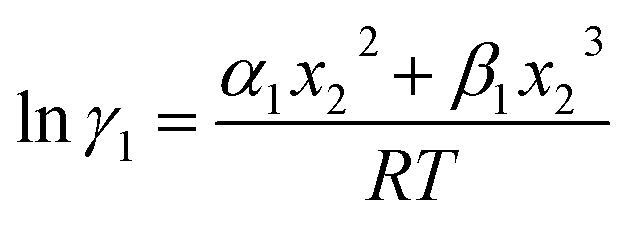
7
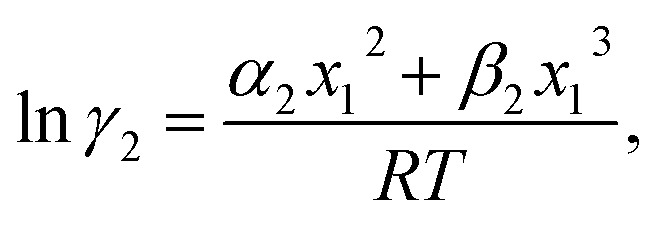
where *α*_1_ = (2*A*_21_ − *A*_12_), *β*_1_ = (2*A*_12_ − 2*A*_21_), *α*_2_ = (2*A*_12_ − *A*_21_), and *β*_2_ = (2*A*_21_ − 2*A*_12_). These parameters are obtained from the activity coefficients of species 1 in species 2 and species 2 in species 1 at infinite dilute condition.^28^ Within the 3-suffix Margules law, at infinite dilution eqn (6) and (7) can be written as ln *γ*^asym^_i/S_ = *A*/*RT*, where *A* is equal to *RT* ln *γ*^∞^ and which is either *A*_12_ (accounts solubility of species 1 in 2) or *A*_21_ (accounts solubility of species 2 in 1), therefore 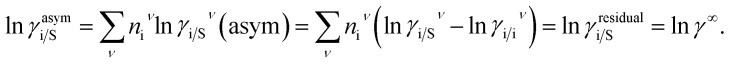 Hence, in COSMOSPACE, one can show that the asymmetric interaction can be well explained using the residual interaction of COSMO-RS^35^ or COSMOSAC^26^ and they are equal to the activity coefficient at infinite dilution (*γ*^∞^) and can be called within the 3-suffix Margules function for a binary mixture. We use the activity coefficient at infinite dilution (*γ*^∞^) calculated using COSMOSAC-2013 (ref. 26) model for all binary mixture solutions used in this work. The same Staverman–Guggenheim (SG)^30,31^ combinatorial term used in the work of Xiong *et al.*^26,36^ has been used here8
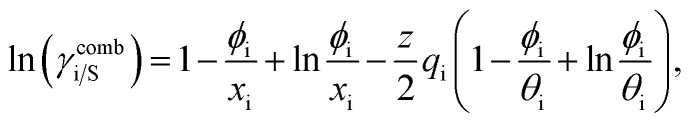
with 
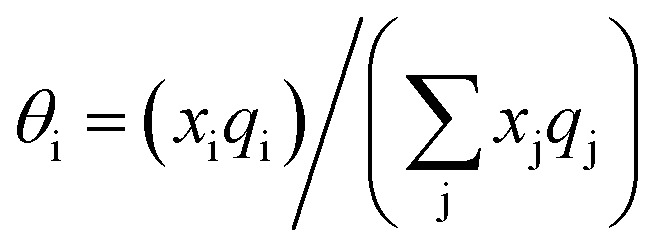
 and 
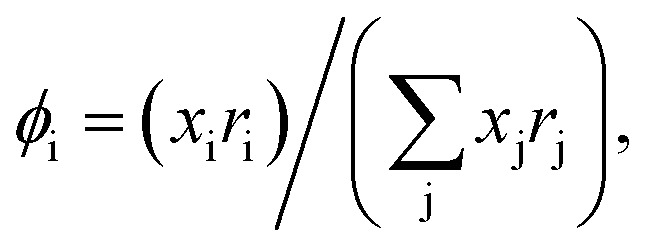
 where *x*_i_ is the mole fraction of component i; *r*_i_ and *q*_i_ are the normalized volume and surface area parameters for species i and *z* is the coordination number equal to 10. The *zq*_i_ represents the number of nearest-neighbor sites to one of the solute and solvent molecules. In this model for combinatorial term only the surface area *q*_i_ has been normalized with *q*_0_ = 79.53 because *r*_0_ will be canceled out in the above equation. After adding the energy terms for combinatorial and asymmetric interactions described above, we obtain the expression for the activity coefficient for species i in solution S and they are for solute (i) and solvent (j) species;9
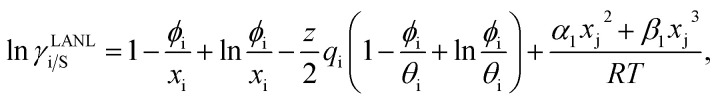
and10
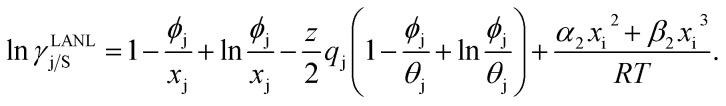


The eqn (9) and (10) are called as “LANL” activity coefficient model in the rest of the paper and in some cases the model is referred as COSMOSAC-LANL model as we imported Margules parameters from COSMOSAC-2013 model. At infinite dilution ((*x*_solute=i_ → 0, *x*_solvent=j_ → 1) the two models COSMOSAC-LANL and COSMOSAC-2013 are connected by

11 ln *γ*(COSMOSAC-LANL) − ln *γ*(COSMOSAC-2013) = ln *γ*(comb).

The details of the derivation has been given in Appendix A. It is noteworthy in this regard that in the COSMOSAC-LANL model the asymmetric interaction has been defined explicitly in terms of 3sM function and the residual interaction has been called implicitly within the 3sM function. To verify the asymmetric solution model, we computed various thermodynamical properties (such as *G*^ex^ and Δ*G*^mix^) and the correlations between ln *γ*_i_, ln *γ*_j_ and 
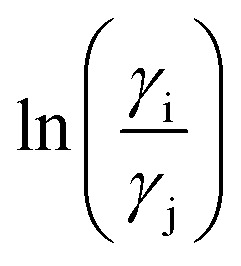
 with varying mole fraction of solute species.^22^ We calculated the total excess Gibbs free energy (*G*^ex^) of the binary system from the equation

12 *G*^ex^ = *RT*(*x*_i_ln *γ*^COSMOSAC-LANL^_i/S_ + *x*_j_ln *γ*^COSMOSAC-LANL^_j/S_),

and the free energy of mixing for the binary mixture using

13 Δ*G*^mix^ = *RT*(*x*_i_ln *x*_i_ + *x*_j_ln *x*_j_ + *x*_i_ln *γ*^COSMOSAC-LANL^_i/S_ + *x*_j_ln *γ*^COSMOSAC-LANL^_j/S_),

where *x*_i_ and *x*_j_ are the mole fractions of solute and solvent molecules in a binary mixture and ln *γ*^COSMOSAC-LANL^_i/S_ and ln *γ*^COSMOSAC-LANL^_j/S_ are the activity coefficients of solute and solvent molecules, respectively obtained from the eqn (9) and (10). The solubility has been calculated using the following equation^37^14
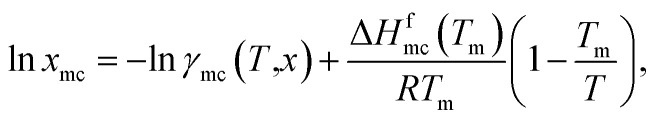
where, *x*_mc_ is the mole fraction of the metal complex in the liquid state, Δ*H*^f^_mc_ is the heat of fusion and *T*_m_ is the melting temperature of the metal complex. For a particular metal complex Δ*H*_mc_ and *T*_m_ are fixed values, therefore, if we vary solvent, the solubility is a function of activity coefficient of the metal complex in liquid,15
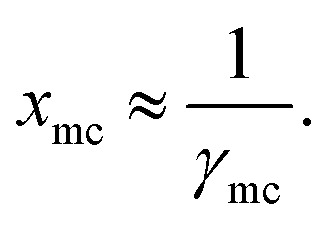


## 3 Computational details

All the COSMO^25^ files for the metal complexes and ionic liquids were generated using the Amsterdam Density functional software version 2016.^26,38^ The geometry optimization was performed using generalized gradient approximation (GGA) with BP exchange correlation functional. The zero order regular approximation (ZORA) method was used for calculating the relativistic effect. TZP basis set for small frozen core and Becke integration with spline Zlm fit for density fitting have been used in ADF for each molecule followed by the post COSMOSAC calculation already implemented in ADF for the calculation of sigma profile of each molecule. It is to be noted that the parameters used in the COSMOSAC-2013 (ref. 26) model are not optimized for transition metals and hence for their complexes and for ionic liquids. So, in our calculations we used the parameters which were optimized for the simple organic molecules and their systems in the ADF software for COSMOSAC-2013 model. The details of quantum COSMO settings have been given in the paper on COSMOSAC-2013 model of Xiong *et al.*^26^ The unrestricted calculation considering spin polarization has been done only for the transition metal complexes as a new addition in the already stated COSMO setting in the work of Xiong *et al.* All ionic liquids have been treated as a single intact ionic liquid molecule during quantum calculation since the total charge of each cation and anion is always less than 1 due to the intermolecular charge transfer between cation and anion reported in the work of Kirchner *et al.*^39,40^ According to their studies, ionic liquid with different clusters sizes of unity charges is erroneous because significant charge transfer between the ionic liquid ions reduces their total charge. To double check, we calculated the σ profile for 10 ionic liquids by first treating them as (case I) single intact molecule and then as (case II) separate cation and anion moieties and calculated their σ profiles. σ profile (*p*_σ_) is defined as the probability distribution for finding a segment of the COSMO surface with charge density *σ*.^41^ In most cases, significant differences in their σ profile were observed for the different type of treatments (case I) and (case II) of ionic liquids shown in Fig. 8 in Section 4.2. Notice that the first step in the COSMOSAC calculation is geometry optimization. For each COSMOSAC calculation, to ensure the minimization in optimized geometry, we also calculated the analytical frequency to check any presence of imaginary frequency in the calculated spectra for the 11 ionic liquids used in this study. The optimized geometry after this calculation has been used for the COSMOSAC calculation implemented in ADF for the version 2013.^26^ The calculated analytical frequencies for the 10 room temperature ionic liquids for which we propose the solubility model have been given in Fig. S-1† and for [Tea][BF_4_] has been given in Fig. S-4(a) in the ESI.† The details of the quantum calculation setting for the analytical frequency calculations have been given in Table 1. The initial conformers of V(acac)_3_ (Fig. 10) metal complex were selected randomly, except the Conf_5_ (taken from CSD^42^), followed by pre-optimization using UFF method and geometry optimization has been done for each conformer followed by the COSMOSAC calculation.

**Table 1:** The details of frequency calculations

Basis set	TZP
Frozen core	Small
Task	GO & frequency calculation
XC	Becke–Perdew
Frequency	Analytical
Numerical quality	Excellent

## 4 Results

### 4.1 σ profile of metal complexes

**Fig. 2:**
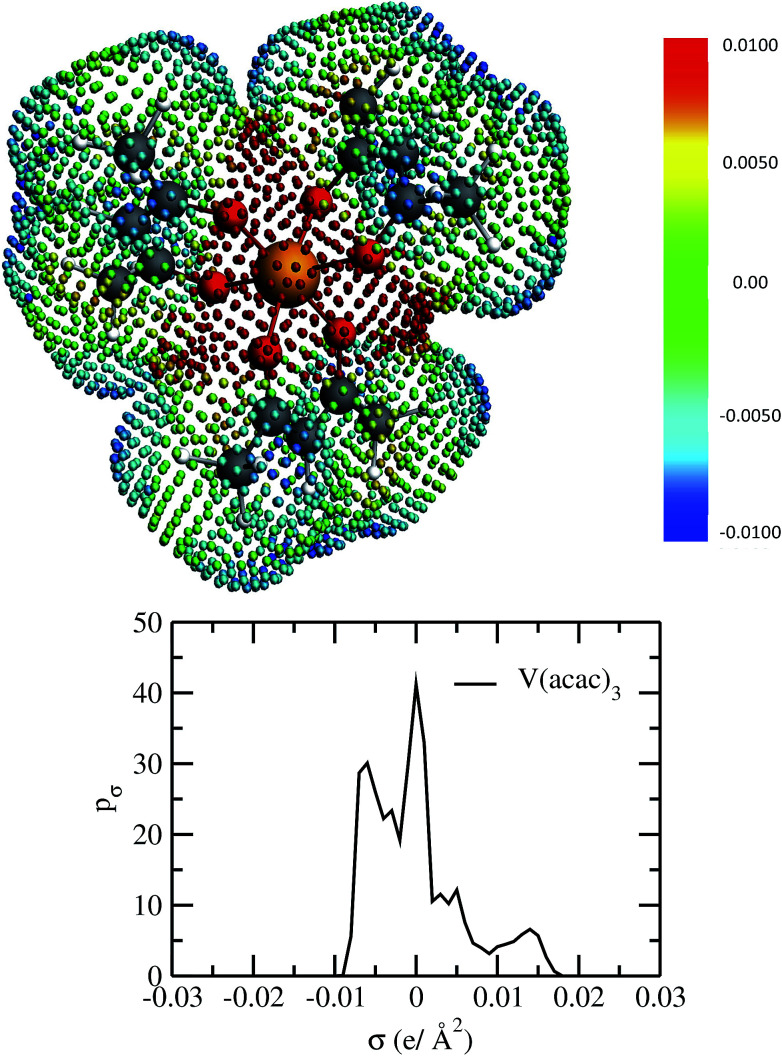
The COSMO surface charge points of V(acac)_3_ has been shown above with the color bar representing the COSMO charge density. The corresponding σ profile of V(acac)_3_ has been shown below.

**Fig. 3:**
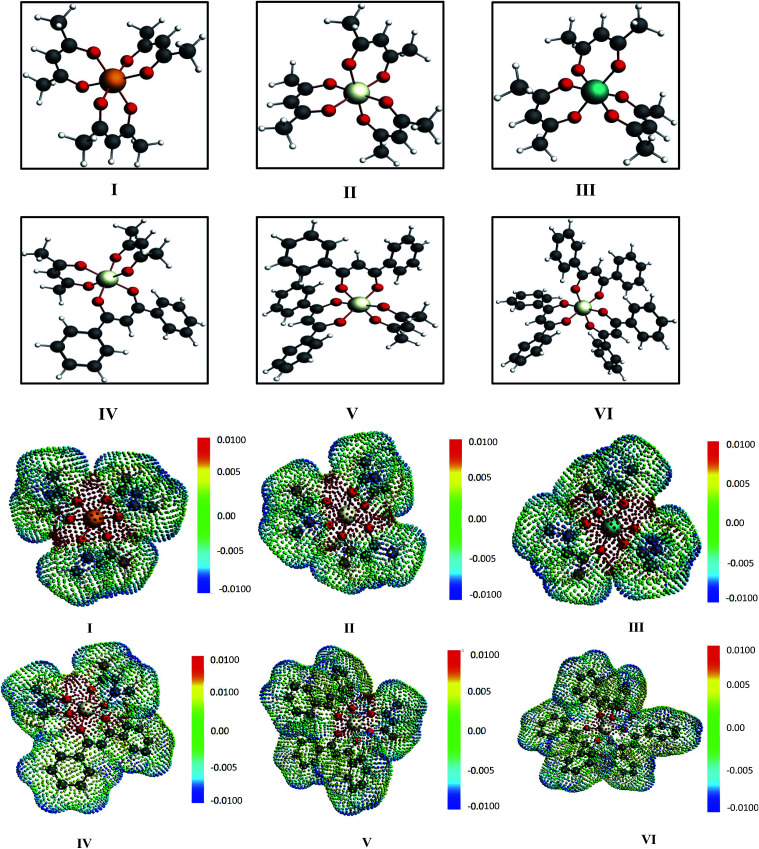
The different metal complexes: (I) tris acetylacetonato vanadium, (II) tris acetylacetonato chromium and (III) tris acetylacetonato manganese (IV) tris(1,3-diphenyl-1,3-propanedionato)chromium, (V) (2,4 pentanedionato)bis(1,3-diphenyl-1,3-propanedionate)chromium and (VI) bis(2,4-pentanedionato)(1,3-diphenyl-1,3-propanedionato)chromium and the corresponding COSMO charge points of all metal complexes have been shown. The initial conformers of I, II, III and IV have been taken from CSD database.^42,46^ The color bar shows the COSMO charge density.

In spite of the lack of COSMOSAC parameters for transition metals in the metal complex molecules of this study one can safely assume that the ligand will play the major role in the solubility of metal complex because it is the ligand the one interacting with the solvent (see Fig. 2 and 3). The effect of the metal is assumed to be *via* its effect on the electronic structure of the ligands. Therefore, we used a group contribution method^43–45^ that assumes that the total σ profile of the metal complex is the summation of the σ profile of each ligand present in the metal complex.

**Fig. 4:**
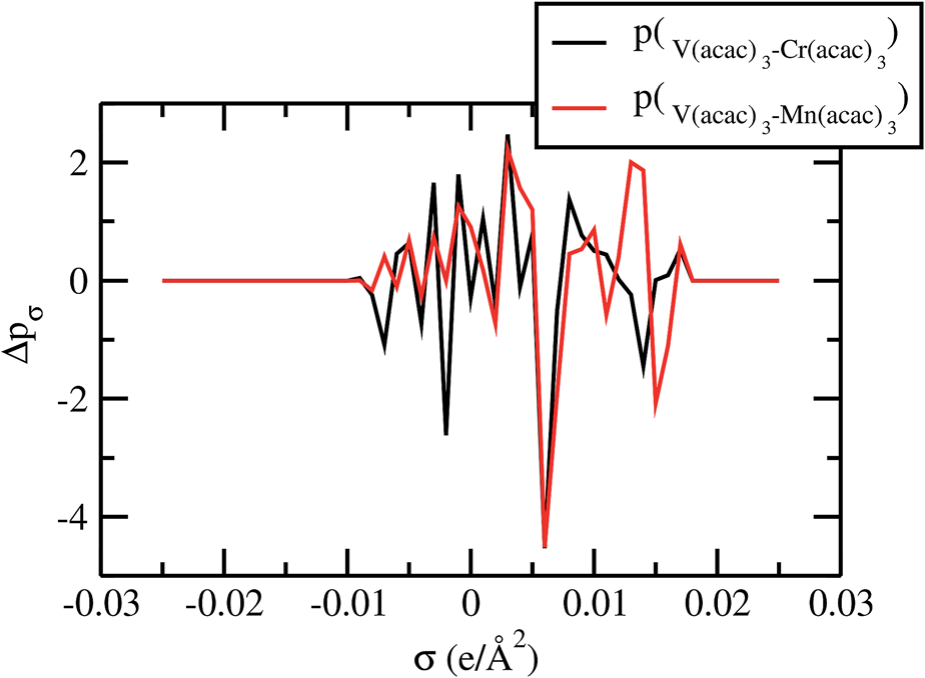
Sigma profile of metal complexes.

**Fig. 5:**
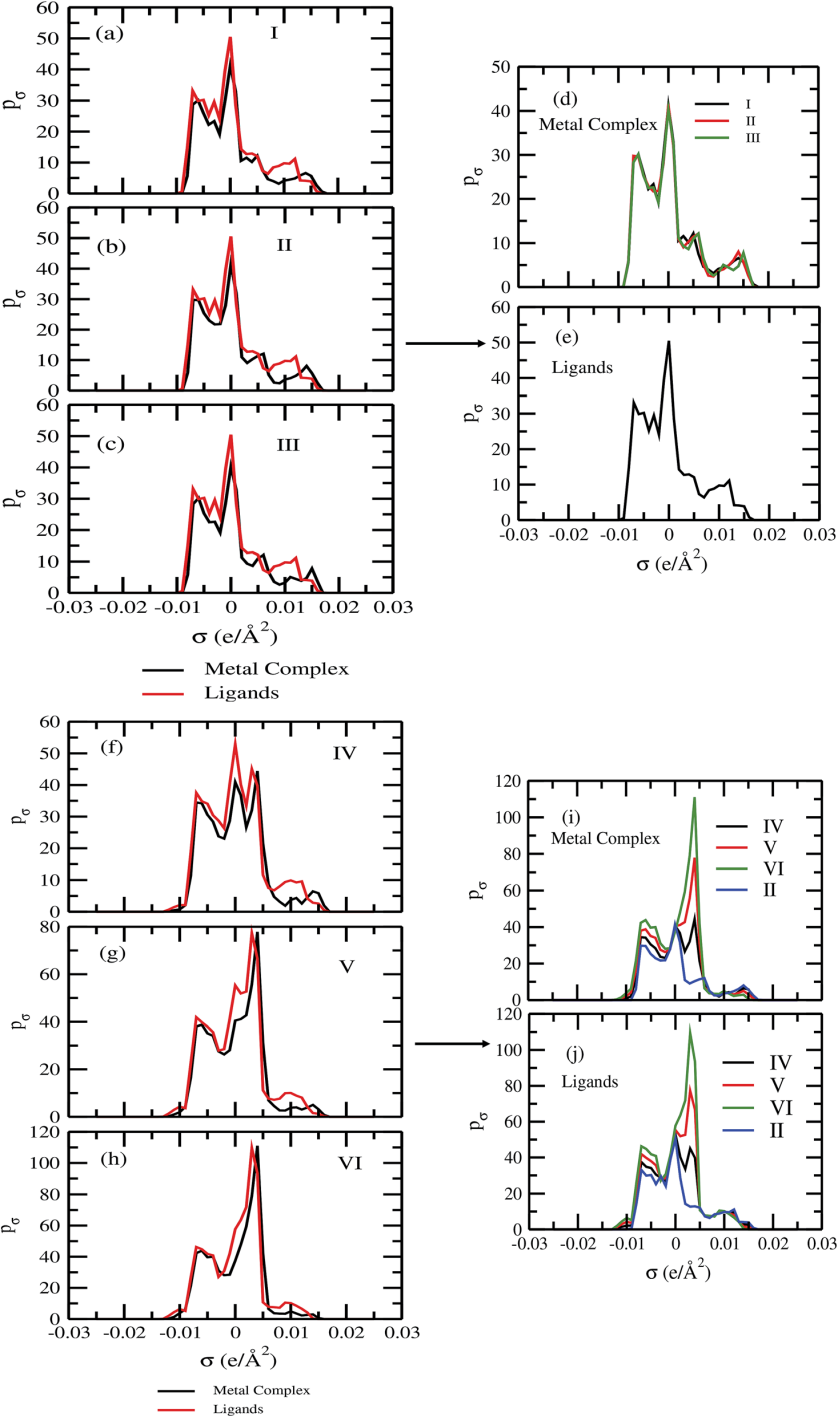
The σ profile of the metal complexes with same ligands with different metal centers and with different ligands with the same metal center.

**Fig. 6:**
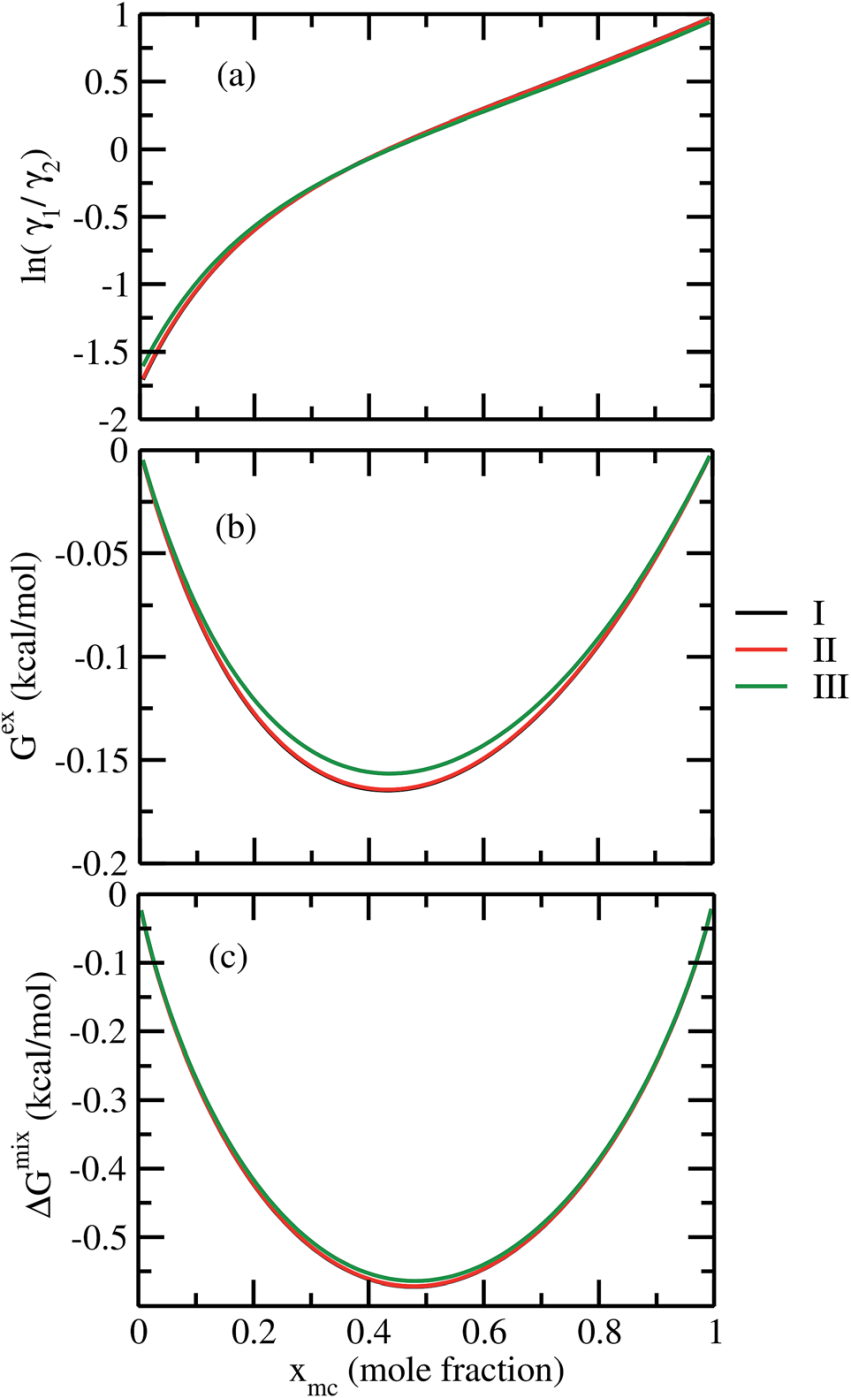
(a) ln *γ*_1_/*γ*_2_, in (b) *G*^ex^ and, in (c) Δ*G*^mix^ have been shown as a function of solute mole fraction for the metal complexes with same ligands but different metal center. The *γ*_1_ and *γ*_2_ are the activity coefficient of the solute and solvent molecule, respectively. The solvent is acetonitrile (ACN).

**Fig. 7:**
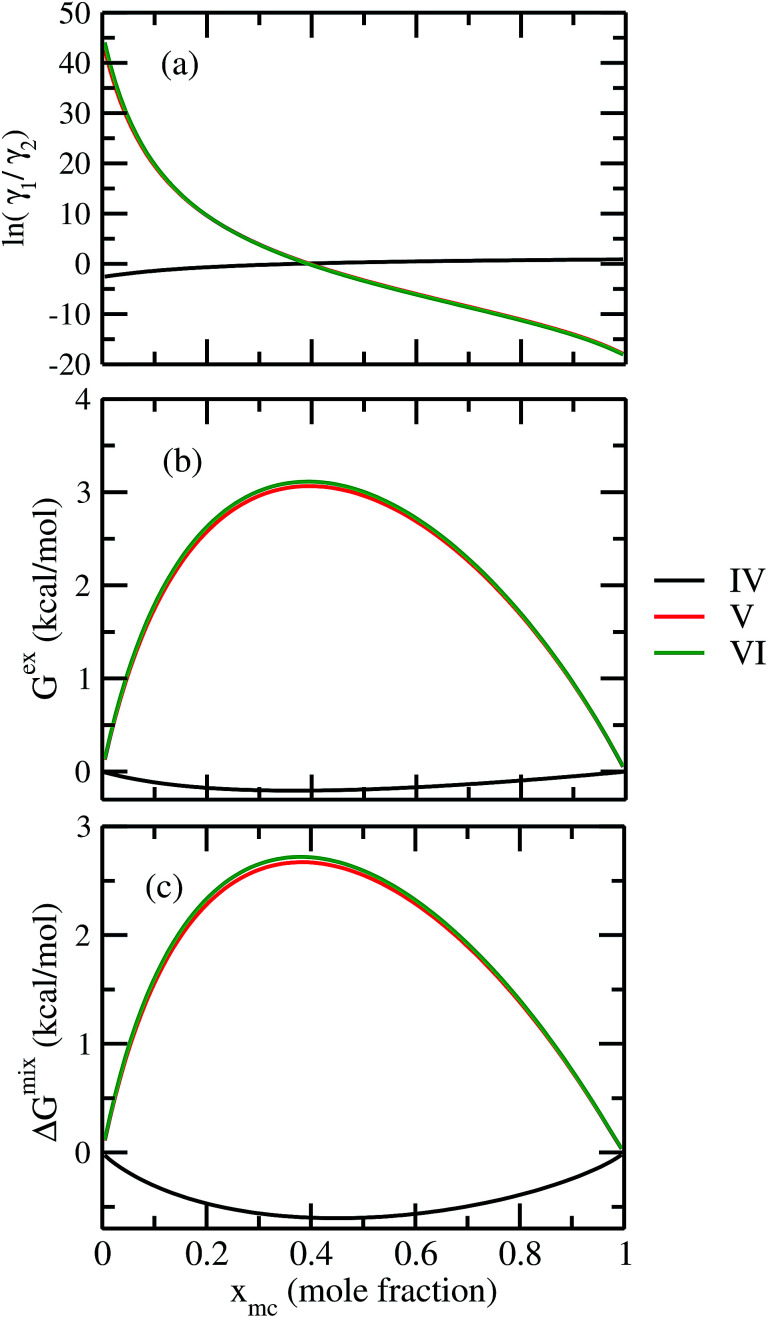
(a) ln *γ*_1_/*γ*_2_, in (b) *G*^ex^ and, in (c) Δ*G*^mix^ have been shown as a function of solute mole fraction for the metal complexes with different ligands but same metal center. The *γ*_1_ and *γ*_2_ are the activity coefficient of the solute and solvent molecule, respectively. The solvent is acetonitrile (ACN).

For our study we follow two families of metal complexes: (a) with same ligands but different metal centers and (b) with different ligands with same metal center. In the first case we studied vanadium, chromium and manganese with acetylacetonate (acac) ligand. In the second case the ligands were derivatives of acac with systematic Ph group substitution coordinating chromium. The structure of the metal complexes and the ligand induced COSMO charge density on the metal center for six different metal complexes studied in this work have been shown in Fig. 3. We also plot the relative differences between the σ profiles of the metal complexes with same type of ligands. In spite of the similar type of σ profiles, we observe a significant difference in their Δ*p*_σ_*vs. σ* plot in Fig. 4. The calculated COSMO surface, volume and molar mass for all metal complexes from I to VI have been given in Table 2. They can be directly correlated with the solubility of a metal complex. The reason for higher solubility in a metal complex can be explained using the σ profile which also present the total COSMO surface of the metal complex. We observed that the metal complexes of almost similar COSMO volumes and surfaces have similar type of σ profiles while the metal complexes with different type of ligands have different σ profiles and their total COSMO volume and surfaces are found to increase with the increase of Ph ligand substitution. As seen in Fig. 5, the metal complex with more bulky ligands (VI) is found to show the highest peak height in its σ profile which also corresponds to the increasing total COSMO surface. In COSMOSAC model, activity coefficient (*γ*_residual_) is calculated from the σ profile and 1/*γ*_residual_ is directly related to the solubility of a compound according to eqn (15). The calculated σ profile of metal complexes I, II, III, IV, V and VI, shown in Fig. 5, have been compared with their experimental^46^ solubility and are found to show that the metal complex having more COSMO surface is less soluble in polar aprotic solvent like acetonitrile (ACN) (*i.e.*, II > IV > V > VI). It is also clear from our study that the metal complex with greater COSMO charge density on the metal center is found to show more solubility in the organic solvent such as acetonitrile. Our qualitative prediction of solubility in terms of the σ profile of different metal complexes is consistent with the experimental solubility of those metal complexes in acetonitrile (ACN).^46^ The factors affecting the solubility of a metal complex in non-aqueous media are (i) activity coefficient of a metal complex in a non-aqueous medium, (ii) molar mass, (iii) heat of fusion and, (iv) melting temperature of the metal complex according to eqn (14) and (15). According to the present study higher molar mass means lower solubility since higher molar mass is associated with the less solubility and therefore, high heat of fusion. As shown in Table 2, a metal complex with higher molar mass and bigger total COSMO surface will be less soluble in a non-aqueous medium for the same metal center with different type of electron withdrawing ligands. Also, we observed that the metal complexes with similar type of ligands and similar total COSMO surfaces have almost the same solubility in case of V(acac)_3_ (∼0.6 M (ref. 1) and ∼0.4 M (ref. 46)), Cr(acac)_3_ (∼0.65 M)^46^ and Mn(acac)_3_ (∼0.60 M)^46^ metal complexes, respectively. The qualitative solubility trend observed in our calculations has been explained in Fig. 6 and 7. For the metal with similar type of ligands, the results can be well explained by the ln(*γ*_1_/*γ*_2_) *vs. x*_mc_, *G*^ex^ (kcal mol^−1^) *vs. x*_mc_ and Δ*G*^mix^ (kcal mol^−1^) *vs. x*_mc_ plot. The *γ*_1_ and *γ*_2_ are the activity coefficient of the solute and solvent molecule, respectively. The slight variation in their solubility can be easily observed in their respective plots shown in Fig. 6. The metal complex with more negative *G*^ex^ (kcal mol^−1^) and Δ*G*^mix^ (kcal mol^−1^) has more solubility in acetonitrile (ACN). To explain the solubility trend for the metal complex in presence of the electron withdrawing ligand (such as Ph), we consider the activity coefficient due to the combinatorial term to calculate the ln(*γ*_1_/*γ*_2_), *G*^ex^ (kcal mol^−1^) and Δ*G*^mix^ (kcal mol^−1^). Using the information about the combinatorial term we calculated the ln(*γ*_1_/*γ*_2_) *vs. x*_mc_, *G*^ex^ (kcal mol^−1^) *vs. x*_mc_ and Δ*G*^mix^ (kcal mol^−1^) *vs. x*_mc_ plot for the metal complex with different type of ligands. In Fig. 7, the positive excess free energy for the metal complex V and VI correspond to the lower solubility of them in acetonitrile with respect to the IV. Therefore, our study on σ profile for such metal complexes was not only able to provide a qualitative prediction of the solubility of a metal complex in acetonitrile solvent, it was able to explain the experimental solubility results too even in the absence of metal parameter in the COSMOSAC model. Also, it is noticed from our study that the effect of the entropic term due to the combinatorial interaction is significant enough to address any trend observed in the solubility for metal complexes IV, V and VI in acetonitrile (ACN). For the metal complexes I, II and III, the residual term for the enthalpy and the combinatorial term for the entropy of the system will play a major role in order to address any trend observed in the solubility. Fig. 5, shows a comparison of the σ profiles of the ligands with the σ profiles of the metal complexes. Hence, it can be concluded that one can calculate the segment activity coefficient for a particular metal complex even in the absence of the parameter for the metal center using COSMOSAC model.

**Table 2:** The COSMO surface, volume, molar mass and experimental solubility of different metal complexes

Metal complexes	COSMO volume (Å^3^)	COSMO surface (Å^2^)	Molar mass (g mol^−1^)	Exp. solubility (M) (ref. 1, 46 and 47)
I	387.35	357.68	348.1	0.6 (ref. 1) and 0.4 (ref. 46)
II	383.47	357.32	358.01	0.65 (ref. 46)
III	379.83	353.29	348.98	0.60 (ref. 47)
IV	532.21	468.12	473.01	0.043 (ref. 46)
V	672.93	581.58	597.05	8E10-4 (ref. 46)
VI	818.12	697.31	721.01	6E10-5 (ref. 46)

### 4.2 σ profile of ionic liquids and their conformational dependence

**Fig. 8:**
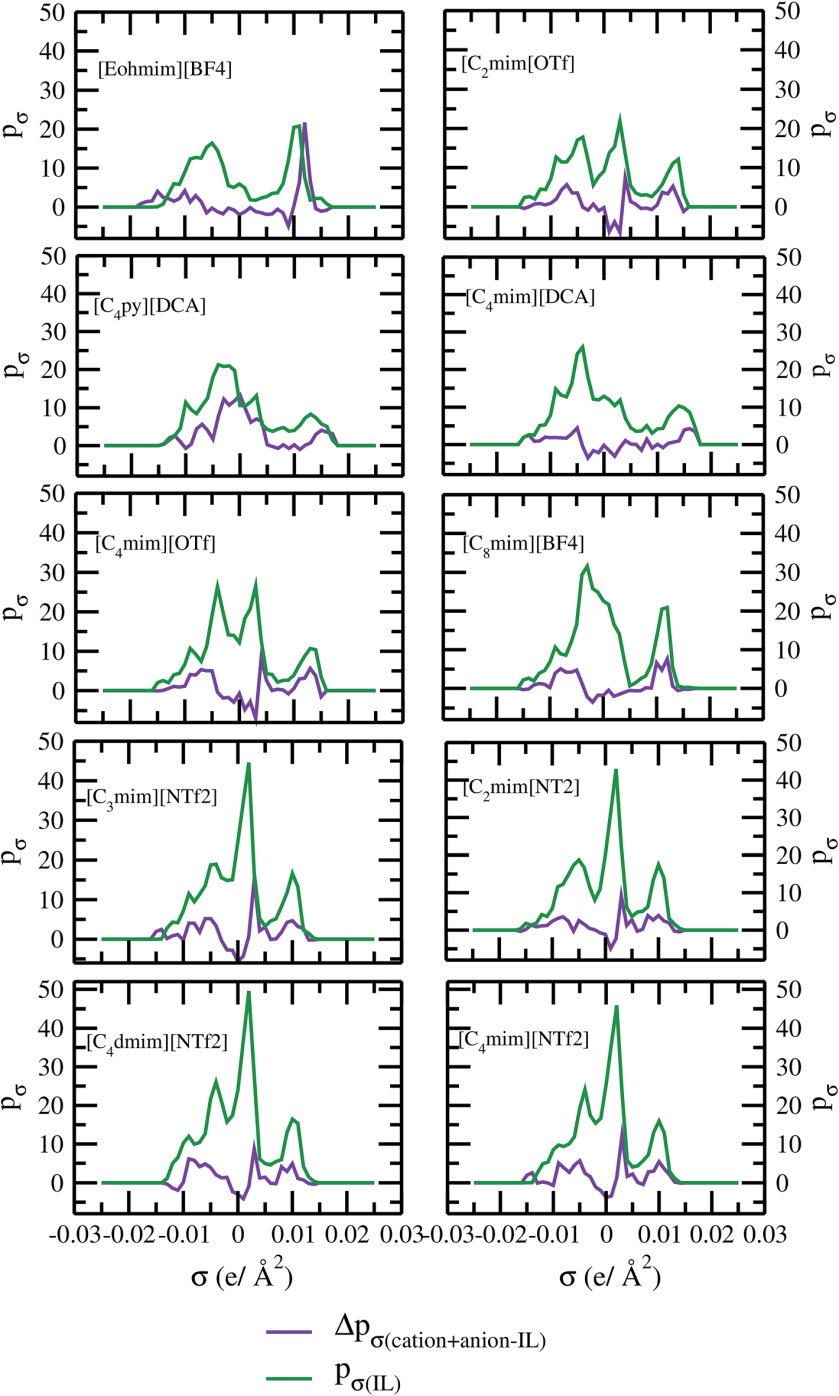
σ profile and their differences for 10 different ionic liquids.

**Fig. 9:**
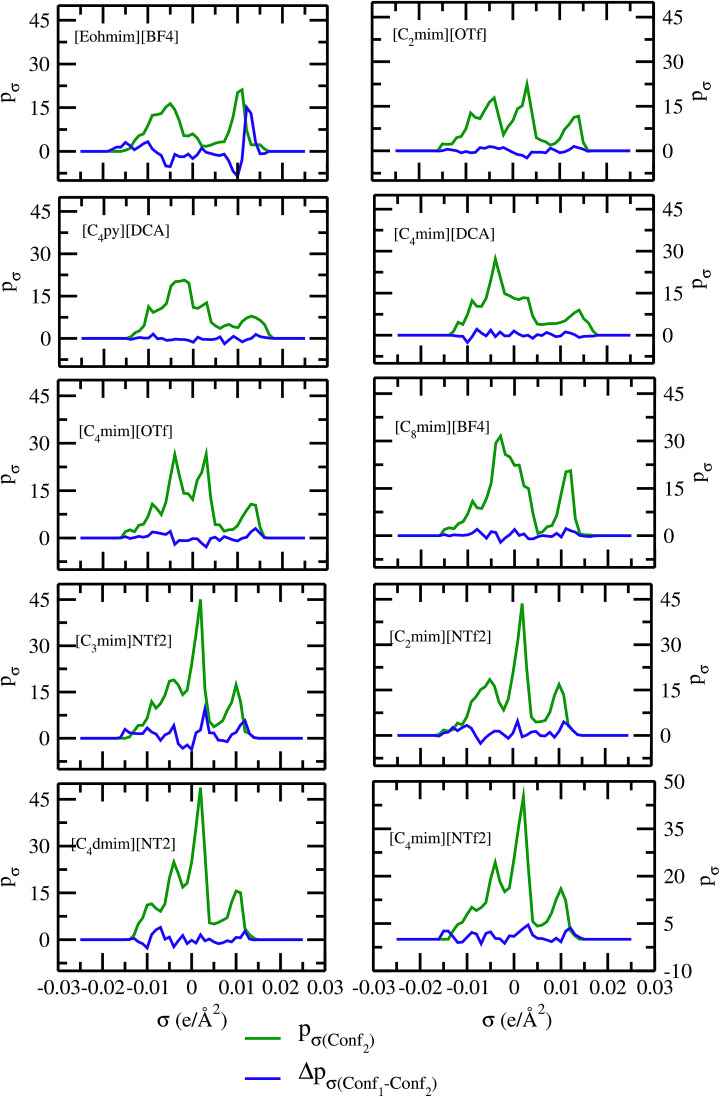
σ profiles and their differences for two different conformations of ionic liquids.

As for metal complexes, COSMOSAC model is missing optimized parameters^26^ for ionic liquids. Ionic liquids have been treated in our calculations as molecule because significant charge transfer between the cation and anion are already reported.^39,40^ We calculated the sigma profiles for ionic liquids as single intact molecule (case I) and as separated cation and anion (case II) for 10 ionic liquids. The result for [Tea][BF_4_] ionic liquid has been shown in Fig. S-4(b).† In all cases, we found significant differences between the σ profiles treated as intact ionic liquid and as solvent separated ions assuming a complete electron transferred from cation to anion shown in Fig. 8. A significant change is observed in the σ profile for the different cations and a particular anion [BF_4_]^−^ for [Eohmim][BF_4_] and [C_8_mim][BF_4_] ionic liquids, when the ionic liquid is treated as single intact molecule instead of being treated as separate entities. These results are shown in Fig. S-6.† In all those cases, the σ profile for the intact ionic liquid indicates that the separate ion model exaggerates the COSMO surface points for the anion shown in black solid line shown in Fig. S-6 in ESI.† The difference between the total σ profile of separate cation and anion and the total σ profile of the neutral ionic liquid is indicative towards the interaction between cation and anion of an ionic liquid shown in Fig. 8. If they did not interact with each other, the difference would be a straight line passing through zero. Also, we observed with the increasing COSMO surface of the ionic liquid the height of σ profile increases for [NTf_2_]^−^ based ionic liquids. It is expected that the bigger molecule with small intermolecular interactions between cation and anion will be responsible for the increasing solubility of a redox active species with minimization of the Gibbs free energy of mixing which should be negative during the dissolution process. Also, treating the ionic liquid as (case I) a single molecular entity and as (case II) separate entities, it will affect the total calculated activity coefficient. In the first case, the system will be treated as a binary mixture but in the second case it will be treated as a ternary mixture in presence of the metal complex and the calculated activity coefficient will be divided by 2 in the second case. A detailed explanation on this has been already given in ref. 38. We calculated the activity coefficient for 10 ionic liquids by considering (case I) and (case II) schemes. The results for 10 ionic liquids are given in Table 3. It is clear from our study that the different treatments of ionic liquid in the COSMO calculation will lead to different results in activity coefficient at infinite dilution. The differences are very prominent which also indicating towards the intermolecular interaction between the cation and anion of an ionic liquid. All 10 ionic liquids studied along with their COSMO surface points have been given in Fig. S-2 and S-3 in ESI.† The similar result for [Tea][BF_4_] has been shown in Fig. S-4(c) in the ESI.† We observed that the different treatment of ionic liquids significantly affect the activity coefficient, which plays an important role in the solubility calculations. The effect of molecular conformations on the σ profile was studied for the ionic liquids and the results for σ profiles have been shown in Fig. 9. No significant changes were observed in the σ profiles when the significant difference in the conformations was observed for all cases. The different conformers of the 10 ionic liquids have been shown in ESI in Fig. S-5.† Also, the σ profiles of the ionic liquids for two different conformers are found to show almost no change in their σ profiles calculated for [NTf_2_]^−^ based ionic liquids shown in Fig. S-7.† Almost no changes in σ profile has been observed for two different conformers except the case where the strong intermolecular hydrogen bond formation occurs between the cation and anion to affect the overall COSMO charge distribution of those ionic liquids (such as [Eohmim][BF_4_]) and hence will affect their σ profile, shown in Fig. 9. However, we observed significant changes in the calculated activity coefficient at infinite dilution for two different conformers because the σ profile results from the COSMO surface, while the activity coefficient is a result of COSMO surface and COSMO volume. The results for activity coefficient at infinite dilution for 10 ionic liquids for two different conformers are given in Table 4 and the two different conformers have been shown in Fig. S-5 in ESI.† We observed that the difference in conformers can be gauged in terms of the difference in activity coefficients. We also compare the bond energy of Conf_1_ (obtained by performing ADF-COSMOSAC-2013 calculation) and Conf_2_ (obtained by performing ADF-COSMOSAC-2013 calculation precede the geometry optimization and analytical frequency calculation in ADF). We observed two different conformers of same bond energy after the COSMO calculation in homogeneous conductor while they were generated in two different ways in this study. However, to propose our model we use Conf_2_ for all 10 ionic liquids for which prior geometry optimization followed by analytical frequency calculation has been done. Though we observed two different conformers of same bond energy, the calculated activity coefficient at infinite dilution for two different conformers differ significantly and this happens due to the noticeable change observed in their COSMO volume and surfaces. The results for different treatment of the ionic liquids and the different conformations have been shown in Fig. S-6 and S-7,† respectively.

**Table 3:** The effect of different treatment of ionic liquids on activity coefficient at infinite dilution (*γ*^∞^)

Numbers	Ionic liquids	*γ* ^∞^	
		Molecule	Separate
1	[Eohmim][BF_4_]	33.29	156.14
2	[C_2_mim][OTf]	3.64	3.09
3	[C_4_py][DCA]	2.74	0.94
4	[C_4_mim][DCA]	2.57	2.37
5	[C_4_mim][OTf]	1.43	1.31
6	[C_8_mim][BF_4_]	1.25	0.96
7	[C_3_mim][NTf_2_]	0.58	0.27
8	[C_2_mim][NTf_2_]	0.56	0.32
9	[C_4_dmim][NTf_2_]	0.53	0.31
10	[C_4_mim][NTf_2_]	0.49	0.24

**Table 4:** The effect of different conformations of ionic liquids on activity coefficient at infinite dilution (*γ*^∞^)

Numbers	Ionic liquids	*γ* ^∞^		Bond energy (hartree)	
		Conf_1_	Conf_2_	Conf_1_	Conf_2_
1	[Eohmim][BF_4_]	707.26	33.29	−5.33	−5.32
2	[C_2_mim][OTf]	4.74	3.64	−5.66	−5.66
3	[C_4_py][DCA]	3.08	2.74	−6.59	−6.59
4	[C_4_mim][DCA]	2.36	2.57	−6.71	−6.71
5	[C_4_mim][OTf]	2.33	1.43	−6.85	−6.85
6	[C_8_mim][BF_4_]	1.47	1.25	−8.67	−8.67
7	[C_3_mim][NTf_2_]	0.56	0.58	−7.62	−7.62
8	[C_2_mim][NTf_2_]	0.67	0.56	−7.03	−7.03
9	[C_4_dmim][NTf_2_]	0.59	0.53	−8.82	−8.82
10	[C_4_mim][NTf_2_]	0.42	0.49	−8.21	−8.22

### 4.3 Conformational dependence of σ profile of metal complex

**Fig. 10:**
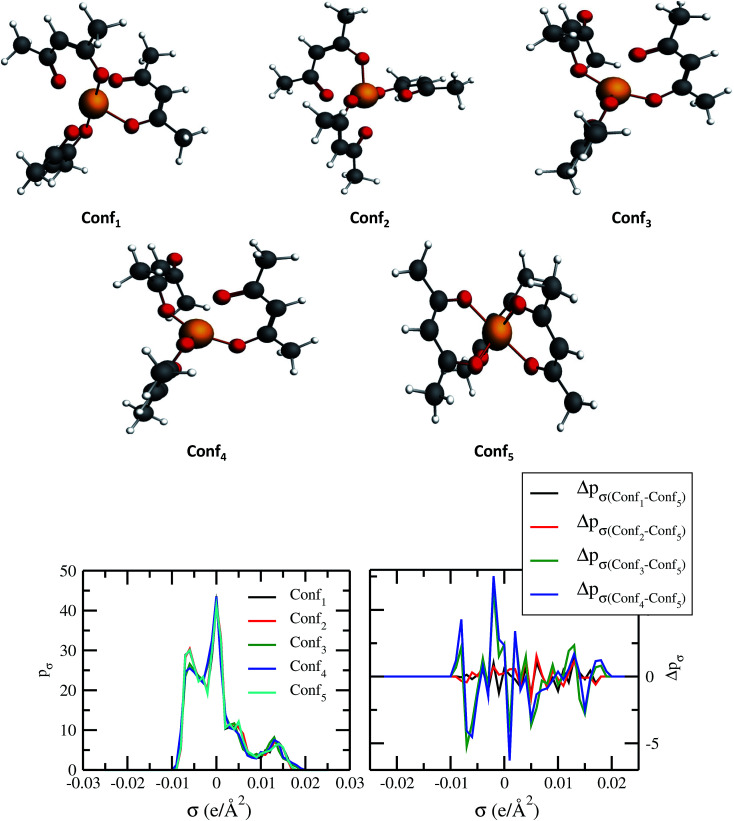
Dependence of σ profiles on the different conformations of V(acac)_3_ metal complex.

The quantum electronic structure based calculation of solubility strongly depends on the conformation of the solute and solvent species. To observe the effect in the σ profile and hence on solubility of metal complexes, we have carried out our calculations for different conformations of V(acac)_3_ metal complex. The details of computation have been given in Section 3. The five different conformations of V(acac)_3_ have been given in Fig. 10 and the σ profiles from all those conformations calculated using ADF-COSMOSAC-2013.^26^ The conformational analysis using Boltzmann weight has been done to get the % populations for each conformer. We found that the % populations for Conf_5_, Conf_2_ and Conf_1_ were 34.71%, 33.03% and 32.27%, respectively. The details of population analysis including Boltzmann weight have been given in Table 5. For the population analysis, we consider the final bond energy resulting from the final COSMOSAC calculation for the five different conformers. To see the effect of conformations on the solubility of the V(acac)_3_ metal complex in short alkyl chain ionic liquid such as [Tea][BF_4_] (commonly used ionic liquid in redox flow cell)^1^ and long alkyl chain ionic liquid such as [C_4_mim][NTf_2_], we carried out our calculations for the inverse of the activity coefficient at infinite dilution (1/*γ*^∞^) and solubility of the metal complex in those ionic liquids. Since solubility depends on the heat of fusion of a compound, to see any effect of that on the conformational dependency of solubility, we did our calculations with the different heat of fusions (Δ*H*_f_) available in the literature and melting temperature (*T*_m_ = 460 K)^48,49^ values for V(acac)_3_ in [Tea][BF_4_] and [C_4_mim][NTf_2_]. The solubility calculations have been done for two different heat of fusions (Δ*H*_f_ = 5.68 kcal mol^−1^ and 7.17 kcal mol^−1^)^48,49^ and melting temperature (*T*_m_ = 460 K) for V(acac)_3_.^48,49^ The calculated activity coefficient and solubility from COSMOSAC-LANL model have been given in Table 6. We observed that the solubility varies from 0.0125 to 0.0138 mole fraction for [Tea][BF_4_] and from 0.0193 to 0.0276 mole fraction for [C_4_mim][NTf_2_], for five conformers and for Δ*H*_f_ = 7.17 kcal mol^−1^ (ref. 48) and melting temperature (*T*_m_ = 460 K). For Δ*H*_f_ = 5.68 kcal mol^−1^ (ref. 49) and melting temperature (*T*_m_ = 460 K), the solubility varies from 0.0245 to 0.0272 mole fraction for [Tea][BF_4_] and from 0.0465 to 0.0645 mole fraction for [C_4_mim][NTf_2_] ionic liquid. We observed that the effect of a particular conformer on the solubility increases with increasing solubility from [Tea][BF_4_] to [C_4_mim][NTf_2_] ionic liquid and hence with the decreasing value of heat of fusion of the metal complex. All the solubility calculations have been done at 330 K and 297 K for [Tea][BF_4_] and [C_4_mim][NTf_2_], respectively because [Tea][BF_4_] is not a room temperature ionic liquid.

**Table 5:** Details of population analysis for different conformers of metal complex with Boltzmann weight

Conformers	*G* (hartree)	Δ*G* (hartree)	Δ*G* (kcal mol^−1^)	Mole fraction	Population
Conf_5_	−9.60518	0.0	0.0	0.3471	34.71
Conf_2_	−9.60513	0.0	0.0295	0.3303	33.03
Conf_1_	−9.60511	0.0001	0.0433	0.3227	32.27
Conf_4_	−9.57320	0.0320	20.0569	0.0	0.0
Conf_3_	−9.57149	0.0337	21.1306	0.0	0.0
*x̄*	−9.58957	—	—	—	—
*σ*_E_	0.0078	—	—	—	—
*δ*_E_	0.0035	—	—	—	—
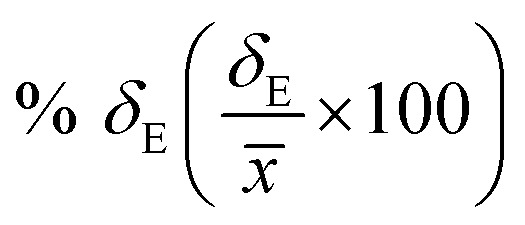	0.037	—	—	—	—

**Table 6:** Conformational dependency of solubility and heat of fusion of V(acac)_3_ in [Tea][BF_4_] and [C_4_mim][NTf_2_] ionic liquids. The melting temperature for all cases is 460 K

Ionic liquids	[Tea][BF_4_]			[C_4_mim][NTf_2_]			
Heat of fusion (Δ*H*_f_)	1/*γ*^∞^	7.17 (kcal mol^−1^)	5.68 (kcal mol^−1^)		1/*γ*^∞^	7.17 (kcal mol^−1^)	5.68 (kcal mol^−1^)
Geometry		Solubility	Solubility			Solubility	Solubility
Conf_1_	0.287	1.30 × 10^−2^	2.63 × 10^−2^		1.83	2.41 × 10^−2^	5.70 × 10^−2^
Conf_2_	0.269	1.25 × 10^−2^	2.45 × 10^−2^		1.44	1.93 × 10^−2^	4.65 × 10^−2^
Conf_3_	0.277	1.29 × 10^−2^	2.54 × 10^−2^		2.06	2.71 × 10^−2^	6.34 × 10^−2^
Conf_4_	0.277	1.29 × 10^−2^	2.54 × 10^−2^		2.11	2.76 × 10^−2^	6.45 × 10^−2^
Conf_5_	0.297	1.38 × 10^−2^	2.72 × 10^−2^		1.92	2.53 × 10^−2^	5.95 × 10^−2^
*x̄*	0.281	1.30 × 10^−2^	2.58 × 10^−2^		1.87	2.47 × 10^−2^	5.82 × 10^−2^
*σ*_E_	0.008	0.00041	0.00074		0.024	0.0003	0.00067
*δ*_E_	0.003	0.0002	0.0003		0.011	0.00014	0.00030
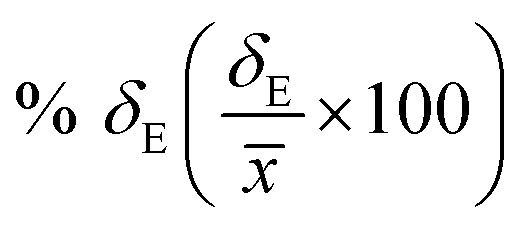	1.06	1.54	1.16		0.59	0.57	0.52

Average property has been constructed for multiple configurations using standard average technique. The uncertainty (*δ*_E_) has been calculated using standard error of mean (SE)16
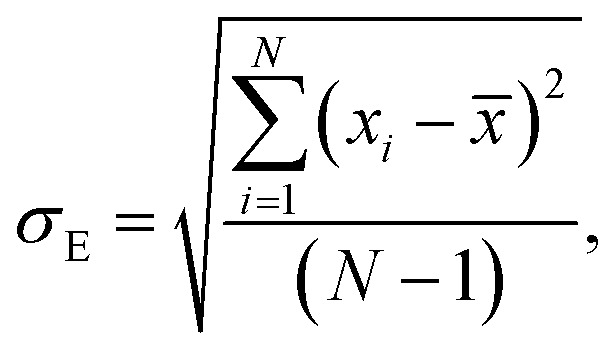
17
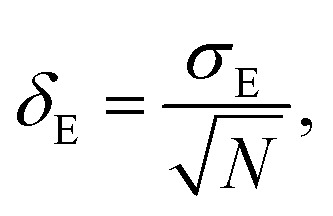
where, *σ*_E_ is the standard deviation, *N* is the sample size, *x*_*i*_ is the *i*th value of a property and *x̄* is the mean and *δ*_E_ is the standard error of mean (SEM). The calculated average solubility for [Tea][BF_4_] is 1.30 × 10^−2^ (±0.0002) and 2.58 × 10^−2^ (±0.0003) for Δ*H*_f_ 7.17 and 5.68 kcal mol^−1^, respectively at a particular temperature 460 K. The same for [C_4_mim][NTf_2_] is 2.47 × 10^−2^ (±0.0001) and 5.82 × 10^−2^(±0.0003) for Δ*H*_f_ 7.17 and 5.68 kcal mol^−1^, respectively at a particular temperature 460 K. The average bond energy noticed in this case is −9.58957 (±0.0035) hartree.

## 5 Solubility of metal complex in ionic liquids

**Fig. 11:**
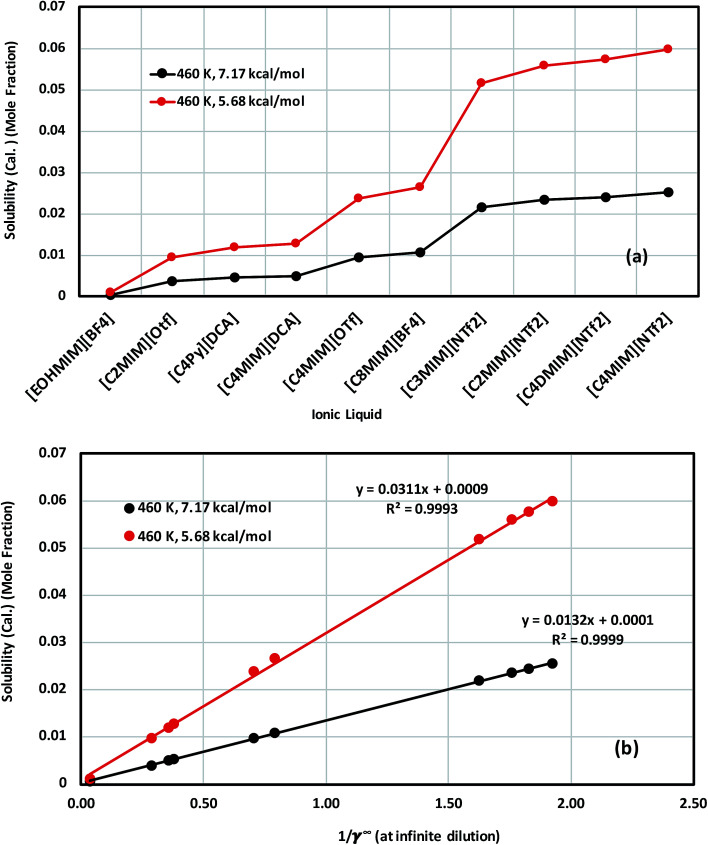
(a) Solubility of V(acac)_3_ in 10 ionic liquids and in (b) the solubility of V(acac)_3_ in 10 ionic liquids is correlated with the activity coefficient at infinite dilution of the metal complex in ionic liquids for two different heat of fusions of V(acac)_3_: Δ*H*_f_ = 7.17 kcal mol^−1^ (black) and Δ*H*_f_ = 5.68 kcal mol^−1^ (red).

The calculated solubility of metal complexes V(acac)_3_ and Cr(acac)_3_ in ionic liquids, and correlation with the inverse of activity coefficient of metal complexes at infinite dilution (1/*γ*^∞^) in ionic liquids are shown in Fig. 11(a), (b), S-8(a) and (b),† respectively. From our theoretical calculations, we found that the solubility of these metal complexes will increase with increasing size of cation and anion of an ionic liquid. For all solubility calculations for V(acac)_3_, we used melting temperature 460 K (ref. 48 and 49) and heat of fusion values 5.68 kcal mol^−1^ (ref. 49) and 7.17 kcal mol^−1^ (ref. 48) for eqn (14). In the case of ideal solvation *γ*_mc_ = 1, the percentage error in solubility is 2.12% for heat of fusion 7.17 kcal mol^−1^ and 4.31% for heat of fusion 5.68 kcal mol^−1^ calculated using eqn (14). We repeated similar calculations for Cr(acac)_3_ in ionic liquids for different heat of fusions and melting temperatures already reported in the literature.^48–53^ We found a similarity with the results already obtained for V(acac)_3_. The solubility calculation for Cr(acac)_3_ has been given in the ESI in Fig. S-8.†

The effect of different heat of fusion and melting temperature has been looked at thoroughly for V(acac)_3_ (Conf_5_) in [Tea][BF_4_] and [C_4_mim][NTf_2_], respectively. We observed that for the average heat of fusion of 6.43 (±1.17) kcal mol^−1^, the average solubility of V(acac)_3_ is 2.05 × 10^−2^ (±0.0037) in [Tea][BF_4_] and 4.24 × 10^−2^ (±0.008) in [C_4_MIM][NTf_2_], respectively at a particular melting temperature. The same calculations have been done for both V(acac)_3_ and Cr(acac)_3_ in 10 ionic liquids have been given in Table 7. The average property (*x̄*), standard deviation (*σ*_E_) and standard error of mean (*δ*_E_) have been calculated following the eqn (16) and (17) in Section 4.

**Table 7:** The solubility of V(acac)_3_ and Cr(acac)_3_ in 10 ionic liquids at different melting temperature and heat of fusion. (I) [Eohmim][BF_4_], (II) [C_2_mim][OTf], (III) [C_4_py][DCA], (IV) [C_4_mim][DCA], (V) [C_4_mim][OTf], (VI) [C_8_mim][BF_4_], (VII) [C_3_mim][NTf_2_], (VIII) [C_2_mim][NTf_2_], (IX) [C_4_dmim][NTf_2_] and (X) [C_4_mim][NTf_2_]

Quantity	I	II	III	IV	V	VI	VII	VIII	IX	X	Δ*H*_f_ (kcal mol^−1^)	*T*_m_ (K)
**V(acac)**_**3**_												
—	4.53 × 10^−4^	3.82 × 10^−3^	4.79 × 10^−3^	5.12 × 10^−3^	9.59 × 10^−3^	1.07 × 10^−2^	2.16 × 10^−2^	2.33 × 10^−2^	2.42 × 10^−2^	2.54 × 10^−2^	7.17	460
—	1.11 × 10^−3^	9.53 × 10^−3^	1.19 × 10^−2^	1.27 × 10^−2^	2.38 × 10^−2^	2.64 × 10^−2^	5.16 × 10^−2^	5.59 × 10^−2^	5.74 × 10^−2^	5.97 × 10^−2^	5.68	460
*x̄*	7.83 × 10^−4^	6.68 × 10^−3^	8.36 × 10^−3^	8.93 × 10^−3^	1.67 × 10^−2^	1.85 × 10^−2^	3.66 × 10^−2^	3.96 × 10^−2^	4.08 × 10^−2^	4.25 × 10^−2^	6.43	460
*σ*_E_	0.0004	0.0030	0.0037	0.0040	0.0075	0.0083	0.0164	0.0177	0.0182	0.0190	2.87	—
*δ*_E_	0.0001	0.0012	0.0015	0.0016	0.0030	0.0034	0.0067	0.0072	0.0074	0.0078	1.17	—

**Cr(acac)**_**3**_												
—	3.96 × 10^−4^	3.29 × 10^−3^	4.15 × 10^−3^	4.44 × 10^−3^	8.09 × 10^−3^	8.89 × 10^−3^	1.69 × 10^−2^	1.89 × 10^−2^	1.90 × 10^−2^	1.98 × 10^−2^	6.78	489
—	3.86 × 10^−4^	3.21 × 10^−3^	4.05 × 10^−3^	4.33 × 10^−3^	7.90 × 10^−3^	8.68 × 10^−3^	1.65 × 10^−2^	1.85 × 10^−2^	1.85 × 10^−2^	1.94 × 10^−2^	6.86	487
—	1.83 × 10^−4^	1.52 × 10^−3^	1.92 × 10^−3^	2.05 × 10^−3^	3.74 × 10^−3^	4.11 × 10^−3^	7.88 × 10^−3^	8.85 × 10^−3^	8.88 × 10^−3^	9.31 × 10^−3^	8.12	481.9
—	1.62 × 10^−4^	1.34 × 10^−3^	1.69 × 10^−3^	1.81 × 10^−3^	3.31 × 10^−3^	3.63 × 10^−3^	6.97 × 10^−3^	7.83 × 10^−3^	7.86 × 10^−3^	8.24 × 10^−3^	8.12	489
—	1.32 × 10^−4^	1.09 × 10^−3^	1.38 × 10^−3^	1.48 × 10^−3^	2.69 × 10^−3^	2.96 × 10^−3^	5.69 × 10^−3^	6.39 × 10^−3^	6.42 × 10^−3^	6.73 × 10^−3^	8.4	490
—	1.27 × 10^−4^	1.05 × 10^−3^	1.32 × 10^−3^	1.42 × 10^−3^	2.59 × 10^−3^	2.84 × 10^−3^	5.47 × 10^−3^	6.14 × 10^−3^	6.16 × 10^−3^	6.46 × 10^−3^	8.57	486
*x̄*	2.31 × 10^−4^	1.92 × 10^−3^	2.42 × 10^−3^	2.59 × 10^−3^	4.72 × 10^−3^	5.18 × 10^−3^	9.90 × 10^−3^	1.11 × 10^−2^	1.11 × 10^−2^	1.17 × 10^−2^	7.8	487.15
*σ*_E_	7.37404 × 10^−5^	0.00061	0.00078	0.00083	0.0015	0.0017	0.0031	0.0035	0.0035	0.0037	0.46	0.83
*δ*_E_	3.01044 × 10^−5^	0.00025	0.00032	0.00034	0.00062	0.00068	0.0013	0.0014	0.0014	0.0015	0.19	0.34

**Fig. 12:**
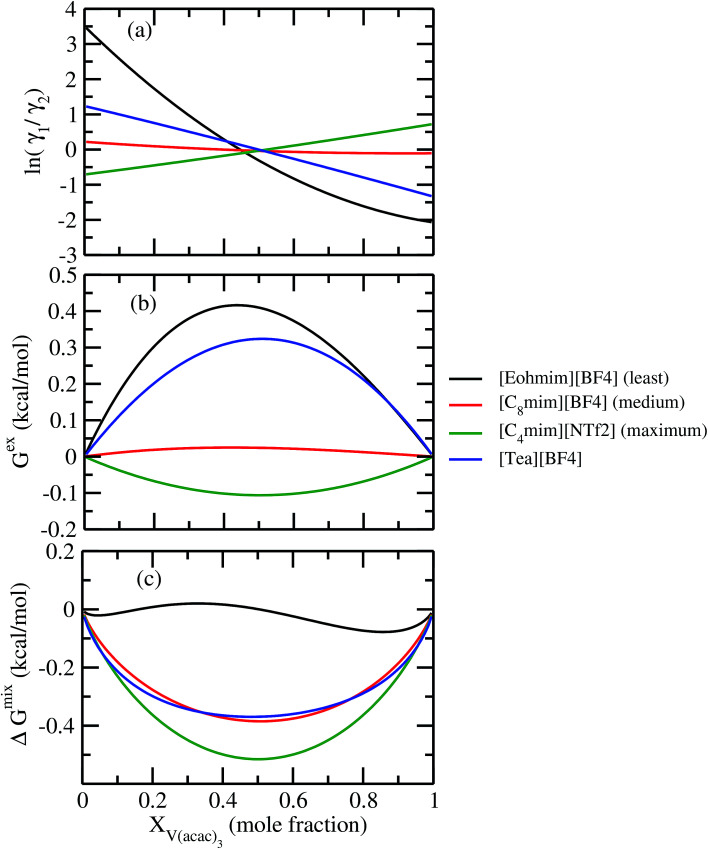
(a) ln *γ*_1_/*γ*_2_, in (b) *G*^ex^ and, in (c) Δ*G*^mix^ have been shown as a function of solute mole fraction at 297 K.

The ionic liquids containing [NTf_2_]^−^ anion were found to be more suitable solvents for redox active species. In the predicted solubility scale in this study, the ionic liquids containing [NTf_2_]^−^ show higher solubility than those having [BF_4_]^−^, [OTf]^−^ and [DCA]^−^ anions. This can be correlated with the bigger size of the ionic liquid as seen in the corresponding σ profile of those ionic liquids in Fig. 8. The bigger ionic liquid will have the maximum peak height in their σ profile due to the higher COSMO surface. On the cation side, the imidazolium and pyrrolidinium cation are found as suitable ionic liquids to increase the solubility of metal complex in redox flow cell. Our theoretical predictions are in good agreement with the experimental work already reported in the work of Katayama *et al.*^13,15–18^ and Ejigu *et al.*^54^ According to their studies, it is already known that the ionic liquid containing imidazolium ring and [NTf_2_]^−^ anion is the suitable non-aqueous solvent medium for redox active species containing transition metal center. We calculated *G*^ex^ and Δ*G*^mix^ for three different ionic liquids with solubility order: (i) least ([Eohmim][BF_4_]), (ii) medium ([C_8_mim][BF_4_]) and (iii) maximum ([C_4_mim][NTf_2_]) to justify our solubility results of V(acac)_3_ in those ionic liquids. In Fig. 12(a), the model was able to capture the asymmetric interaction present in the binary mixture solutions, the monotonic linear relation corresponds to the symmetric nature of the solution whereas the non linear interaction corresponds to the asymmetric nature of the metal complex and ionic liquid mixture solution. According to Fig. 12(b) and (c), the calculated *G*^ex^ and Δ*G*^mix^ for [C_4_mim][NTf_2_] ionic liquid is more negative with respect to [C_8_mim][BF_4_] and [Eohmim][BF_4_] and thus will be responsible for the higher solubility of V(acac)_3_ in [C_4_mim][NTf_2_]. Also, it is observed in the calculations that the ionic liquid ([Eohmim][BF_4_]) containing strong intermolecular hydrogen bond forming moieties between cation and anion shows the least metal complex solubility. This can be attributed to the high cavitation energy needed to accommodate the metal complex in this solvent and thus will reduce the solubility of metal complex in that ionic liquid and will result positive *G*^ex^ and Δ*G*^mix^. The calculated free energy of mixing (Δ*G*^mix^) and excess free energy (*G*^ex^) for three different ionic liquids showing least, medium and maximum solubility of redox active species V(acac)_3_ are shown in Fig. 12. The symmetric interactions between the V(acac)_3_ and [C_8_mim][BF_4_], V(acac)_3_ and [C_4_mim][NTf_2_] and the asymmetric interaction between the V(acac)_3_ and [Eohmim][BF_4_] are shown in the same Fig. 12(a). This is indicative to the less molecular interaction present during the dissolution process of V(acac)_3_ in [C_4_mim][NTf_2_] and [C_8_mim][BF_4_] than [Eohmim][BF_4_].

**Fig. 13:**
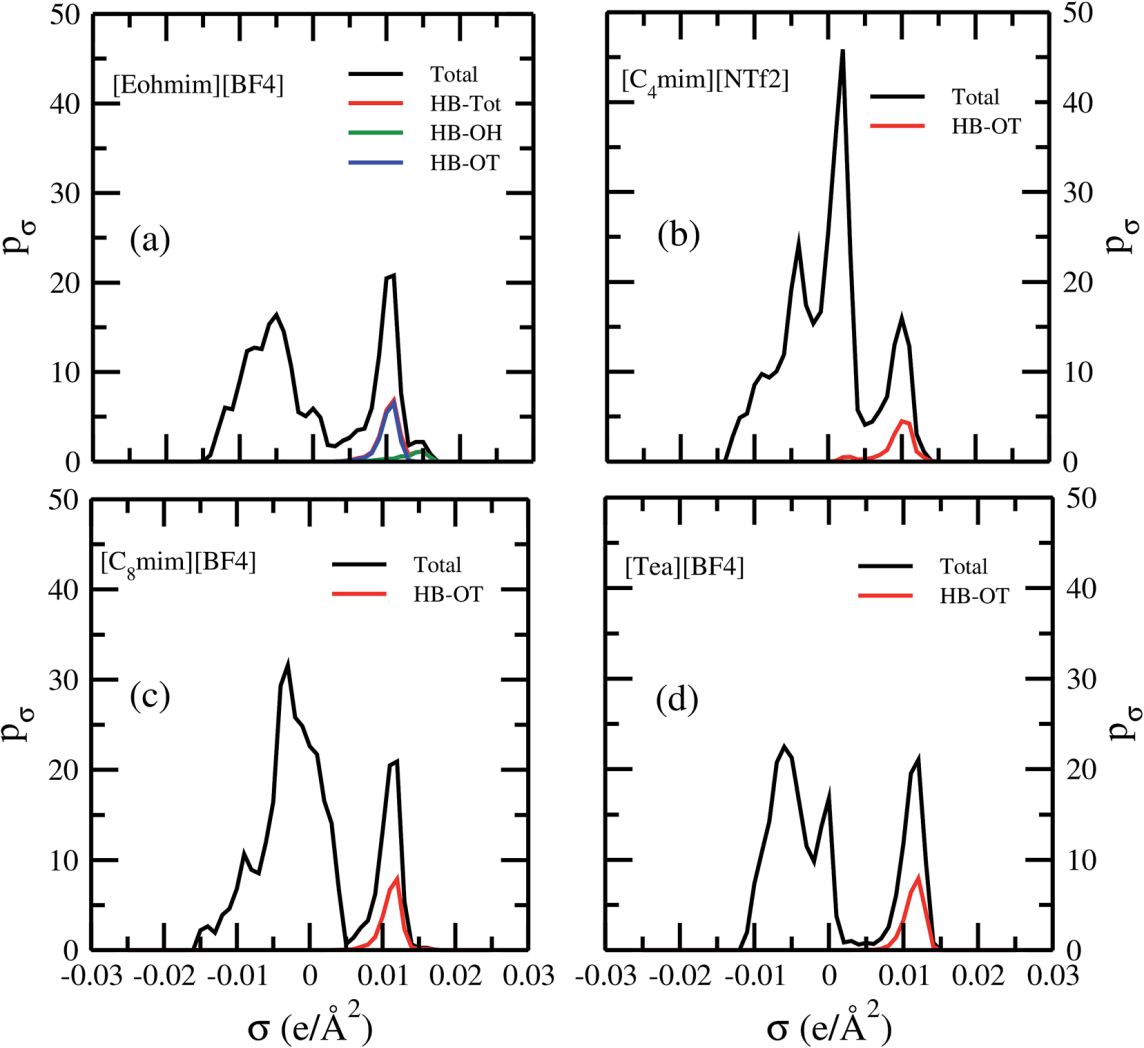
σ profiles for least ([Eohmim][BF_4_]), medium [C_8_mim][BF_4_]), maximum ([C_4_mim][NTf_2_]) and [Tea][BF_4_] metal complex solubility. *p*_σ_(HB) = *p*_σ_(HB–OH) + *p*_σ_(HB–OT) and *p*_σ_(Total) = *p*_σ_(HB) + *p*_σ_(NHB), where *p*_σ_(HB–OH), *p*_σ_(HB–OT) and *p*_σ_(NHB) are the σ profile due to OH group hydrogen bonding, other than OH group hydrogen bonding and non hydrogen bonding group. The details about each term have been given in the work of Xiong *et al.*^26^

Fig. 13 shows the calculated σ profile of three types of ionic liquids categorized as maximum ([C_4_mim][NTf_2_]), medium ([C_8_mim][BF_4_]), least ([Eohmim][BF_4_]) and [Tea][BF_4_] metal complex solubility. COSMOSAC^26,55^ classifies the segment of the COSMO surface into three categories: (a) non hydrogen bonding, (b) hydrogen bonding from OH group and, (c) hydrogen bonding from other than OH group. A Gaussian like function has been considered to express the probability of hydrogen bonding segments18
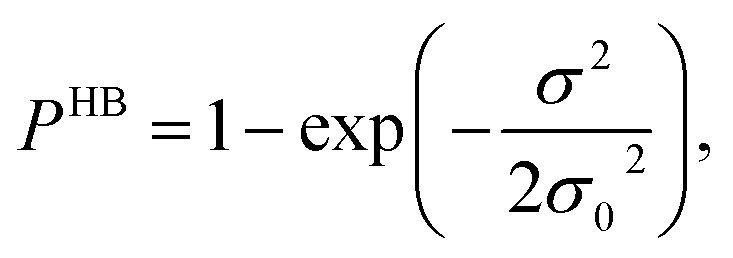
where *σ* is screening charge density and *σ*_0_ is equal to 0.007 e Å^−2^ for the Gaussian distribution. The relation between them is *p*_σ_(HB) = *p*_σ_(OH⋯OH) + *p*_σ_(OH⋯OT) and thus *p*_σ_(Total) = *p*_σ_(HB) + *p*_σ_(NHB), where *p*_σ_(NHB) is the σ profile due to the non-hydrogen bonded group. 
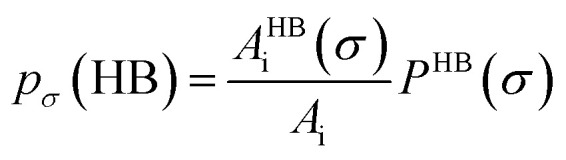
 and 
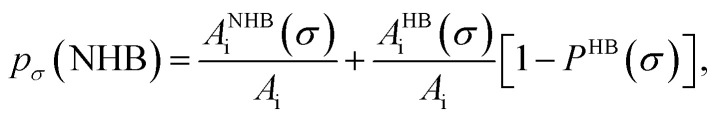
 where *A*_i_ is the COSMO surface. The details are given in the work of Xiong *et al.*^26^ The molecule with the ability to form intermolecular hydrogen bond between hydroxyl (OH) group of the cation and FB group of [BF_4_]^−^ is the least V(acac)_3_ absorber because the physical absorption of V(acac)_3_ molecule in those ionic liquids will result high Gibbs free energy of cavity formation in expense of breaking of intermolecular (H⋯F) hydrogen bonds. Such kind of (OH⋯OT) bond formation has been shown in the Fig. 13; plot (a) by blue line, the smaller amount of (OH⋯OH) has been shown by green line and the total of (OH⋯OT) and (OH⋯OH) has been shown by the red line and the total σ profile *p*_σ_(Total) has been shown by the black line. Also, it has been found from the study that the amount of (HB-OT) bond formation decreases from [Eohmim][BF_4_] to [C_4_mim][NTf_2_] with simultaneous increase of the V(acac)_3_ solubility in them. This can be correlated with the excess Gibbs free energy *G*^ex^ and Gibbs free energy of mixing Δ*G*^mix^ in Fig. 12. The molecule with the ability to form stronger hydrogen bond between the cation and anion is found to show the less solubility of V(acac)_3_ in them. In the other two ILs showing medium and maximum V(acac)_3_ solubility, they have no hydroxyl group (OH). Therefore all those hydrogen bond interactions in those ILs occurs due to the other than hydroxyl group (OT) interactions are weak and these results are reflected in their corresponding σ profiles in Fig. 13; plot (b) and (c). For the comparison, we also show the result for [Tea][BF_4_] in Fig. 13; plot (d). The calculated COSMO surface for the total hydrogen bonded (*p*_σ_(HB) = *p*_σ_(HB–OH) + *p*_σ_(HB–OT)) interaction is 23.95 Å^2^ for [Eohmim][BF_4_], 17.48 Å^2^ for [C_4_mim][NTf_2_], 23.89 Å^2^ for [C_8_mim][BF_4_] and 24.42 Å^2^ for [Tea][BF_4_], respectively.

**Fig. 14:**
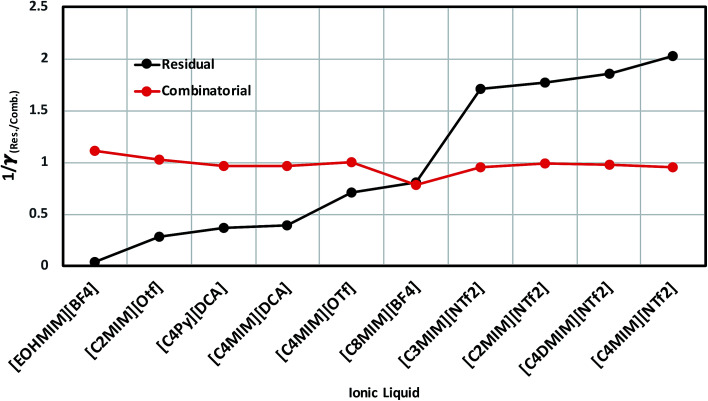
The dependence of residual interaction on the total activity coefficient at infinite dilution and the dependence of combinatorial interaction on the total activity coefficient at infinite dilution.

In order to investigate the effect of residual and combinatorial effect to screen the ionic liquids for a particular metal center such as V(acac)_3_, we performed our calculations for the individual effect of residual and combinatorial term on the solubility of metal complex. We observed that the effect of combinatorial term is very negligible and of the same order for all cases (1/*γ*_comb_ ∼ 1). The residual effect is sufficient enough to screen the ionic liquids for V(acac)_3_. The computed residual and combinatorial terms for V(acac)_3_ in ionic liquids are shown in Fig. 14. Thus, based on our calculations, one will be able to do the solubility prediction of a metal complex such as V(acac)_3_ just by looking at the residual interaction present in the system consisting of transition metal complex and ionic liquid using eqn (15). The 1/*γ*_Res_ is composed of the contribution coming from the residual and dispersion interaction in the COSMOSAC-LANL model in eqn (15) which is shown in Fig. 14. Therefore, the information about the residual interaction will be sufficient enough to screen the ionic liquids for the solubility of metal complex V(acac)_3_ in them.

## 6 Dual solute effect between metal complex and ionic liquid

**Fig. 15:**
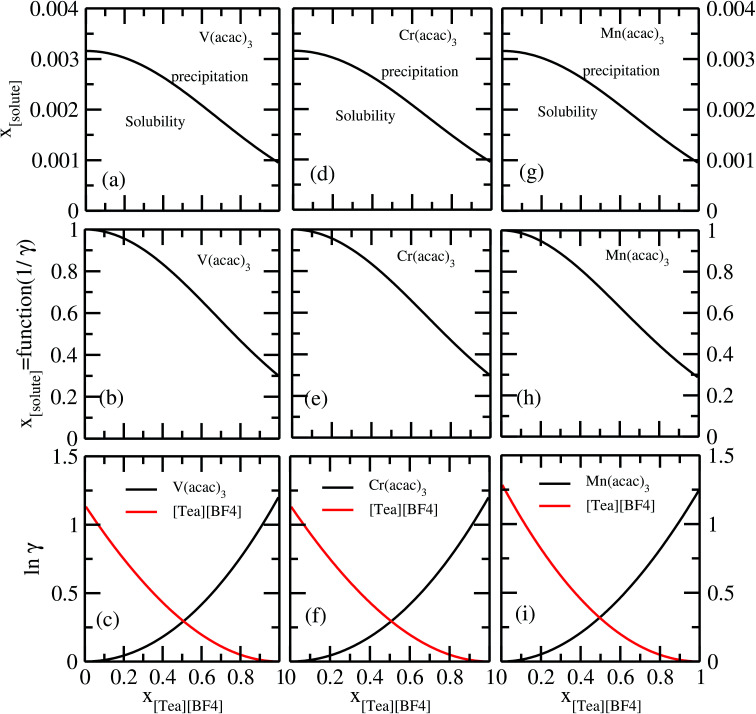
The calculated phase diagrams have been shown in (a) and (b) for V(acac)_3_, in (d) and (e) for Cr(acac)_3_ and in (g) and (h) for Mn(acac)_3_ metal complexes. In (c), (f) and (i), the Gibbs–Duhem relationship for the three binary systems have been shown.

**Fig. 16:**
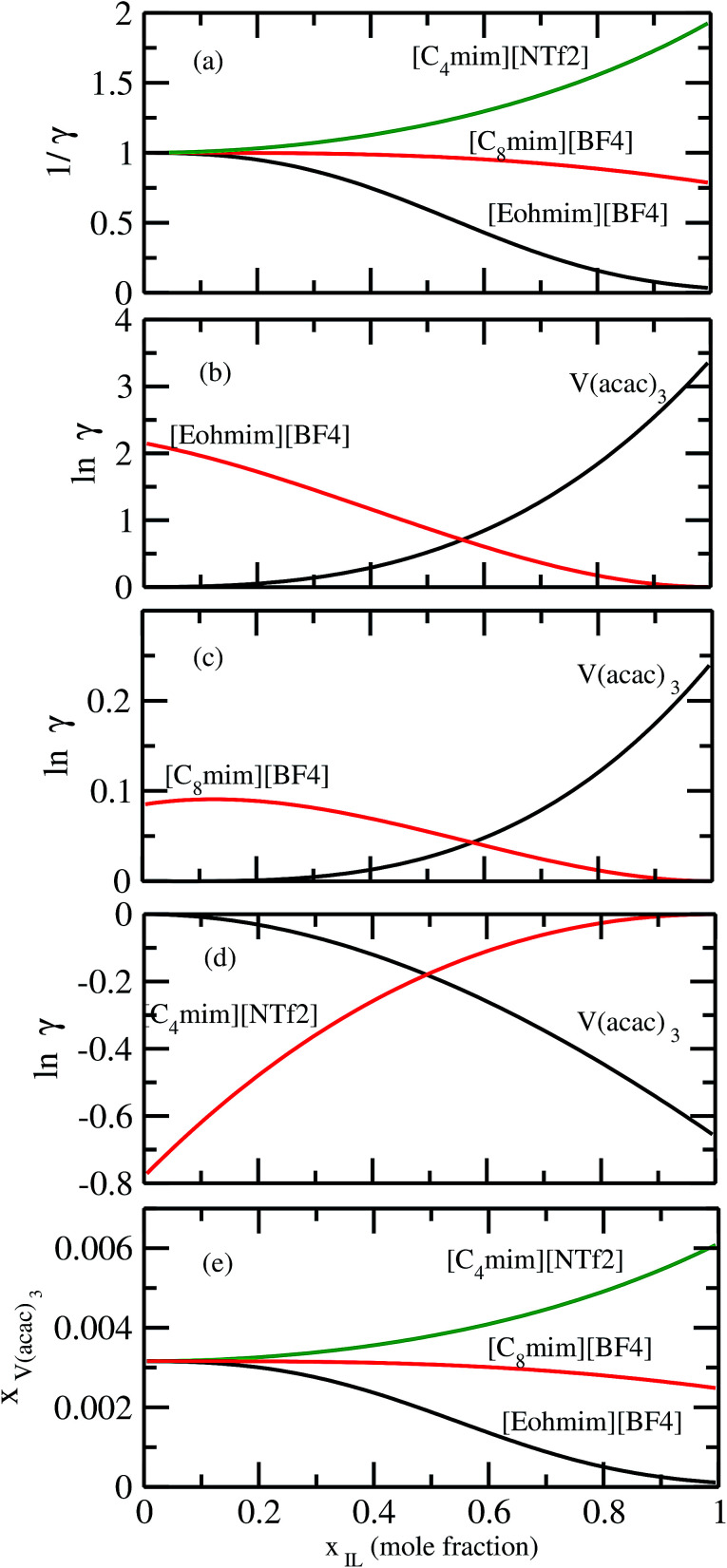
(a) The solubility has been shown as a function of 1/*γ*. The Gibbs–Duhem relationships have been shown for (b) [Eohmim][BF_4_], (c) [C_8_mim][BF_4_] and (d) [C_4_mim][NTf_2_], respectively. (e) The calculated phase diagrams have been shown for all three ionic liquids.

It has been reported that the metal complex will not behave as a sparingly soluble salt in presence of ionic liquid as supporting electrolyte and acetonitrile (ACN) as organic solvent.^1,9^ The experimental studies have been done for ternary systems consist of V(acac)_3_, [Tea][BF_4_] and ACN and the same system with Cr(acac)_3_ and Mn(acac)_3_ metal complex. For all cases, it was observed that the solubility of metal complex decreases with the increase of ionic liquid concentration and this phenomena in the redox flow cell is called dual-solute effect. We have established this effect by considering a binary system consisting of redox active species and ionic liquid for all V(acac)_3_, Cr(acac)_3_ and Mn(acac)_3_ metal complexes. The correlation has been established between the calculated solubility and the inverse of activity coefficient at infinite dilution (1/*γ*^∞^) of metal complexes with the varying mole fraction of the [Tea][BF_4_] ionic liquid. We have found that the calculated phase diagram in absence of the third component is in good agreement with the experimental phase diagram reported at the room temperature^9^ and thus was able to explain the reason behind the experimentally observed dual-solute effect. It is to be noted that the calculations have been done at room temperature to mimic the actual experimental condition used in the article.^1^ From Fig. 15(a), (b), (d), (e), (g) and (h) of all species in the binary mixture, it is found that the dual-solute effect is a consequence of Gibbs–Duhem relationship^28^ between the two species in a binary mixture in the COSMOSAC model. The solubility phase diagrams already found in the work of ref. 9, has been shown in Fig. 15(a), (b), (d), (e), (g) and (h) are in good agreement with the experimental^9^ phase diagram reported for V(acac)_3_, Cr(acac)_3_ and Mn(acac)_3_ metal complexes in [Tea][BF_4_] as supporting electrolyte and ACN as solvent. We also extended our theoretical calculations to the other three ionic liquids showing least, medium and maximum solubility and found that the dual-solute effect favorable to the increasing metal complex solubility in presence of those ionic liquids. Therefore, the duel-solute effect (competing ability for the solubility) observed in case of V(acac)_3_/[Tea][BF_4_]/ACN, Cr(acac)_3_/[Tea][BF_4_]/ACN and Mn(acac)_3_/[Tea][BF_4_]/ACN ternary mixture system is not true or a general case for other ionic liquids. This observation can be explained by computing the Gibbs–Duhem relationship between the solute and supporting electrolyte species and the solubility of the redox active species in them. The reason can be explained by the relationship between the activity coefficient and the mole fractions of the solute and solvent species. For the least and medium absorber ionic liquids, we observed that the activity coefficient of the solute is increasing with the increase of the mole fraction of the ionic liquid while the opposite behavior has been observed for the maximum absorber ionic liquid which can be explained in terms of the symmetric and asymmetric interaction (Fig. 16(a)) between the solute and solvent species and can be directly correlated with the Gibbs–Duhem relationship and solubility (Fig. 16(b), (c), (d) and (e)) observed in each case. Also, these theoretical findings are confirmed by the calculations of ln *γ*_1_/*γ*_2_, *G*^ex^ and, Δ*G*^mix^ as a function of mole fraction of V(acac)_3_ in Fig. 12.

## 7 Conclusion

We have used COSMOSAC-LANL model to predict solubility of metal complexes in ionic liquids and to screen the ionic liquids for a particular metal complex where the metal complex has been treated as a solute and the ionic liquid as a solvent in a binary mixture. Also, we have shown that this model can do qualitative prediction of solubility of different metal complexes with same metal center but with different type of ligands for a particular solvent by comparing their σ profiles using the model. The proposed new model was able to explain the similar experimental solubility results observed in the case of V(acac)_3_, Cr(acac)_3_ and Mn(acac)_3_ in acetonitrile solvent. The bigger size of ionic liquids and metal complexes have been correlated with the solubility of metal complex to get a qualitative idea on solubility trend. We have applied our theoretical model on a series of chromium complexes to get qualitative prediction of their solubility in acetonitrile solvent using their σ profile information because this information is used for the calculation of solubility of the metal complex in the ionic liquid. We found our theoretical results are in good agreement with the experimental results.^46^ According to our calculations, the ionic liquids containing imidazolium cation and [NTf_2_]^−^ are found to show greater solubility of redox active species in them and our results are found to follow the trend already reported in the experimental paper.^13,15–18,54^ We calculated the excess free energy (*G*^ex^) and free energy of mixing (Δ*G*^mix^) for least, medium and maximum absorber of redox active species in ionic liquids and found that the free energy of mixing is less negative for [Eohmim][BF_4_] and [C_8_mim][BF_4_] and thus will reduce the metal complex solubility in them with respect to the solubility of V(acac)_3_ in [C_4_mim][NTf_2_]. The present model was also able to explain the solvation mechanism in those systems.

This model can be used to screen the suitable ionic liquid for a particular metal complex and metal complexes with different ligands and with same metal center against a particular solvent to increase the ionic conductivity, solubility and thus energy density of a redox flow cell where a compatible pairing between metal complex, organic solvent and ionic liquid is an important factor.

## Conflicts of interest

There are no conflicts to declare.

## Appendix A

At infinite dilute condition, when *x*_1_ → 0 and *x*_2_ → 1, the eqn (6) will be reduced to 
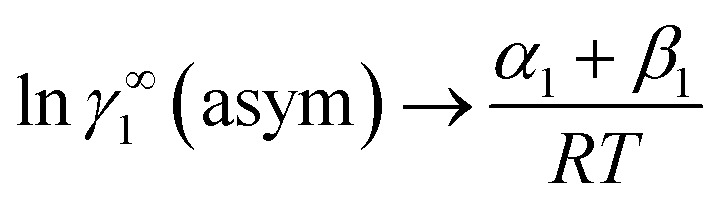
 for solute species (1). Now after adding and substituting the value of (*α*_1_ + *β*_1_) in the expression of ln *γ*^∞^_1_, one will get 
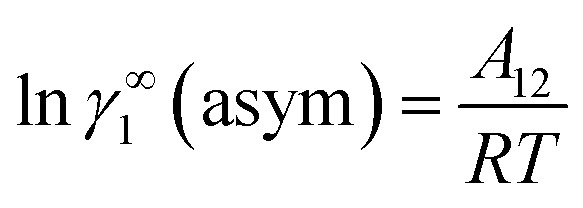
 and hence,

19 *A*_12_ = *RT*ln *γ*^∞^_1_(asym).

Similarly, when *x*_2_ → 0 and *x*_1_ → 1, the eqn (7) will be reduced to 
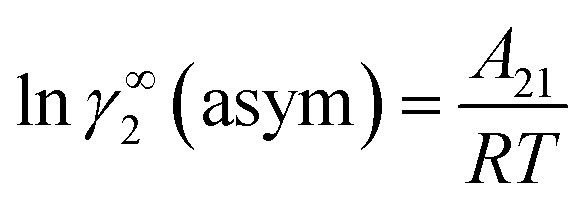
 for the solvent species (2) and therefore,

20 *A*_21_ = *RT*ln *γ*^∞^_2_(asym).

Therefore, at infinite dilution, one can calculate Margules parameters from the activity coefficients at infinite dilution from the above two equations. Since, in this article the Margules parameters are called from the activity coefficient at infinite dilution calculated using COSMOSAC-2013 model, therefore one can write21
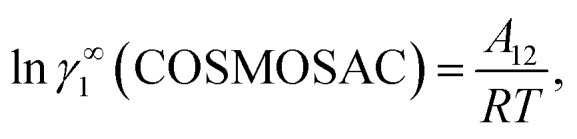
and22
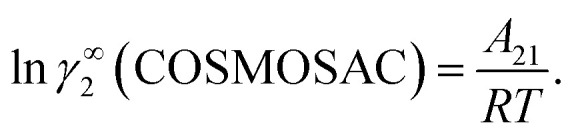


Substituting the value of *A*_12_ and *A*_21_ in eqn (19) and (20), one will get,

23 ln *γ*^∞^_1_(asym) = ln *γ*^∞^_1_(COSMOSAC),

and

24 ln *γ*^∞^_2_(asym) = ln *γ*^∞^_2_(COSMOSAC).

Since, we know that

25 ln *γ*(COSMOSAC-2013) = ln *γ*(res) + ln *γ*(comb) + ln *γ*(dis).

Therefore,

26 ln *γ*(asym) = ln *γ*(res) + ln *γ*(comb) + ln *γ*(dis).

Now, our asymmetric model is,

27 ln *γ*(COSMOSAC-LANL) = ln *γ*(comb) + ln *γ*(asym).

Substituting ln *γ*(asym) in the above equation, one will get,

28 ln *γ*(COSMOSAC-LANL) = 2ln *γ*(comb) + ln *γ*(dis) + ln *γ*(res).

Therefore, subtracting eqn (25) from eqn (28), one will get the relationship between the two models which is valid at infinite dilution and that is

29 ln *γ*(COSMOSAC-LANL) − ln *γ*(COSMOSAC-2013) = ln *γ*(comb).

## Supplementary Material

RA-009-C9RA04042K-s001
