# Supplementary material for: Solubility model of metal complex in ionic liquids from first principle calculations
Source: RSC Adv. 2019 Jun 12;9(32):18506–26. doi: 10.1039/c9ra04042k (PMC9064736; doi:10.1039/c9ra04042k)
Supplement: RA-009-C9RA04042K-s001 [file RA-009-C9RA04042K-s001.pdf]

Electronic Supplementary Material (ESI) for RSC Advances.
This journal is © The Royal Society of Chemistry 2019

Supporting Information

Solubility model of metal complex in ionic liquids from first principle calculations

Anwesa Karmakar*†, Rangachary Mukundan‡, Ping Yang*† and Enrique R. Batista*†

†Theoretical Division, Los Alamos National Laboratory, Los Alamos 87545

‡MPA-11 Division, Los Alamos National Laboratory, Los Alamos 87545

*E-mail: anwesak@lanl.gov; anwesa.karmakar@gmail.com; pyang@lanl.gov; erb@lanl.gov

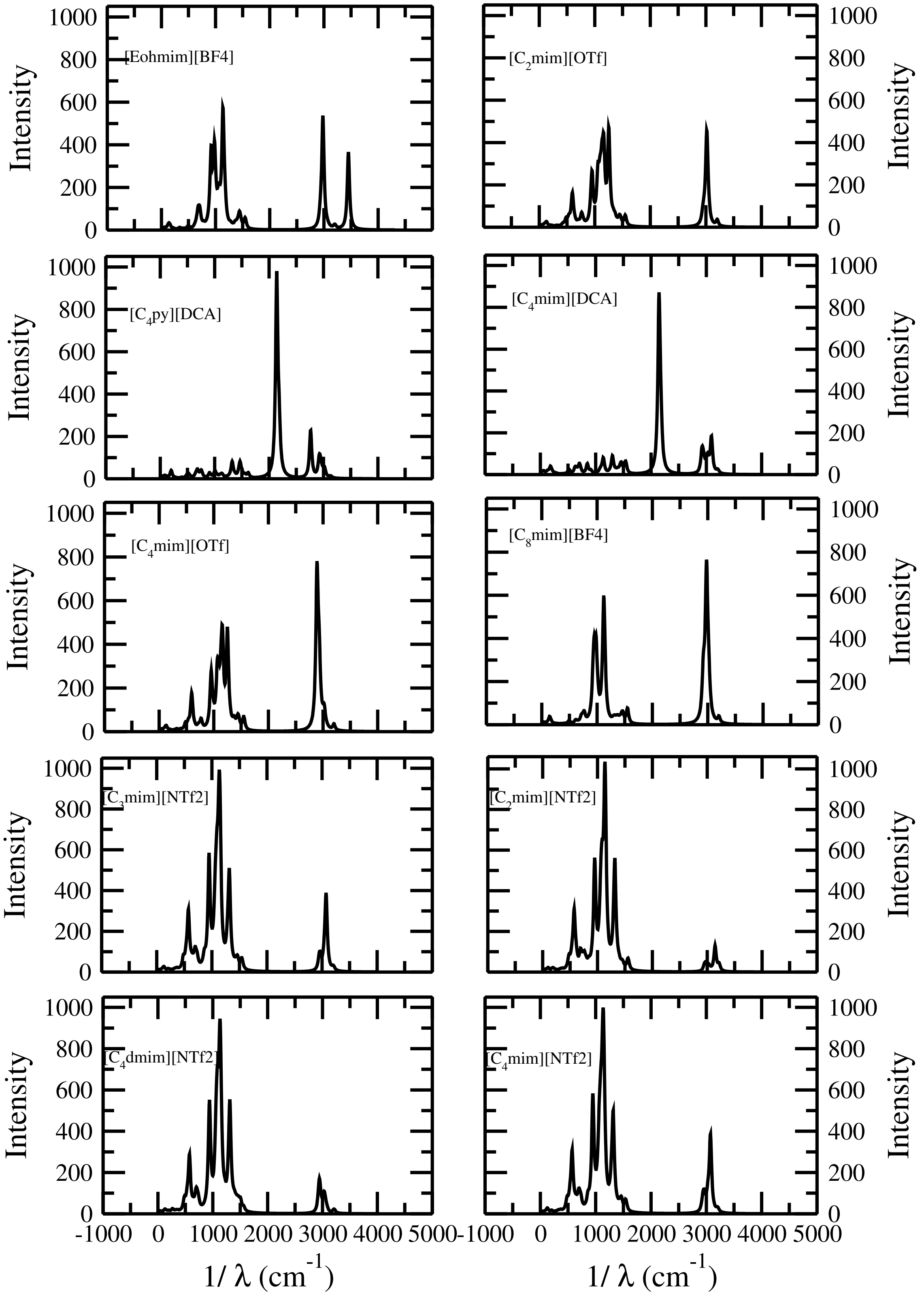

Figure. S 1: Analytical frequency of selected ionic liquids at room temperature.

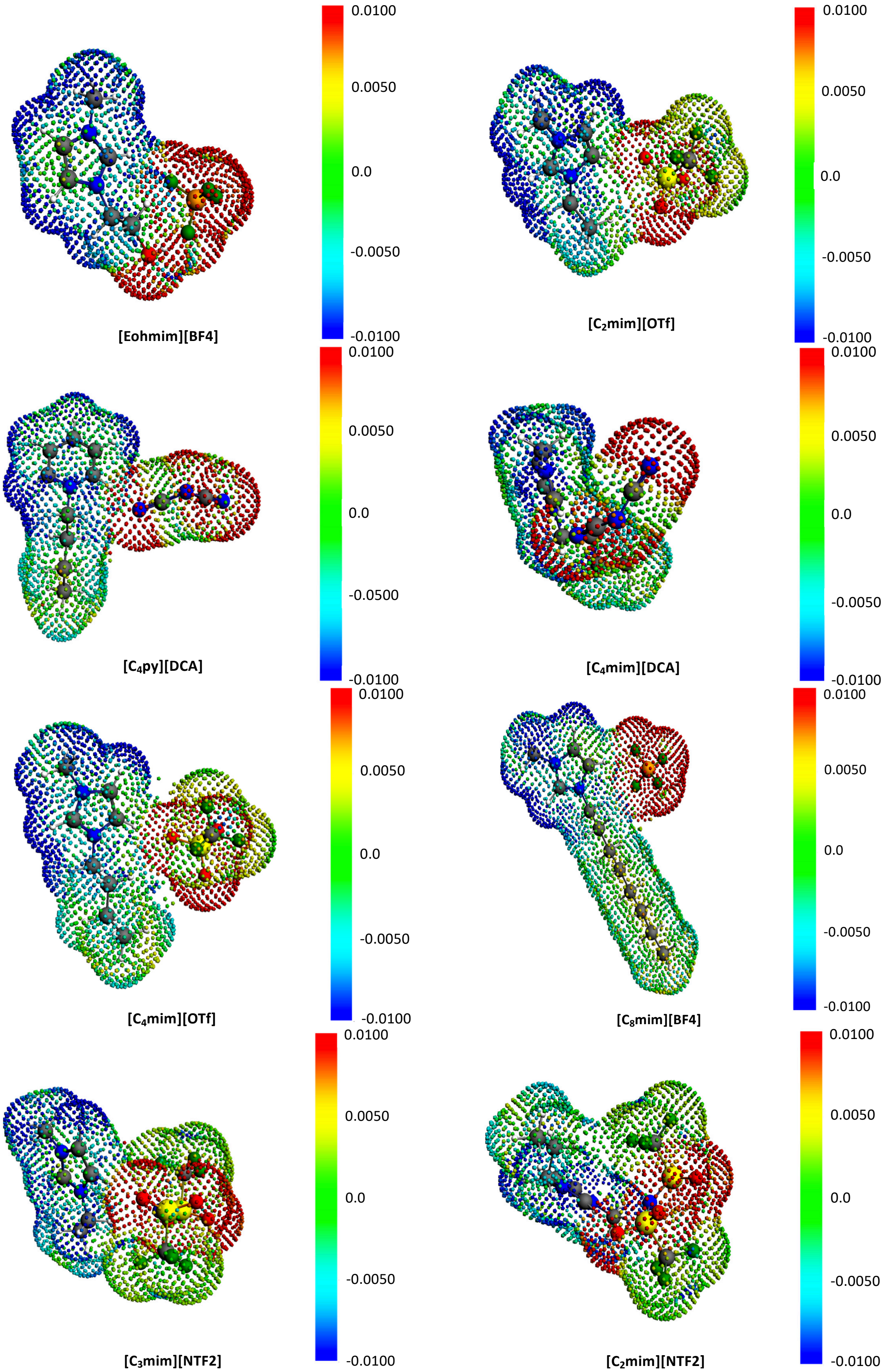

Figure. S 2: COSMO surface points of 10 ionic liquids

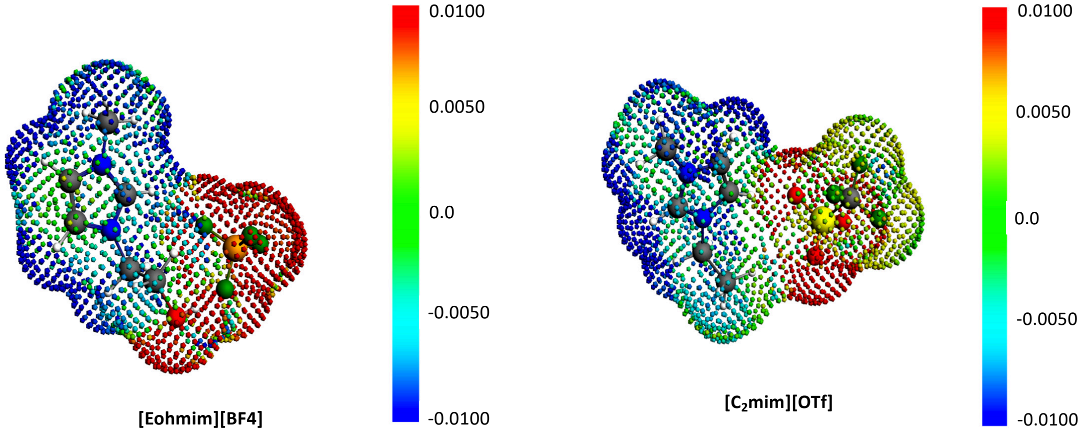

Figure. S 3: COSMO surface points of 10 ionic liquids

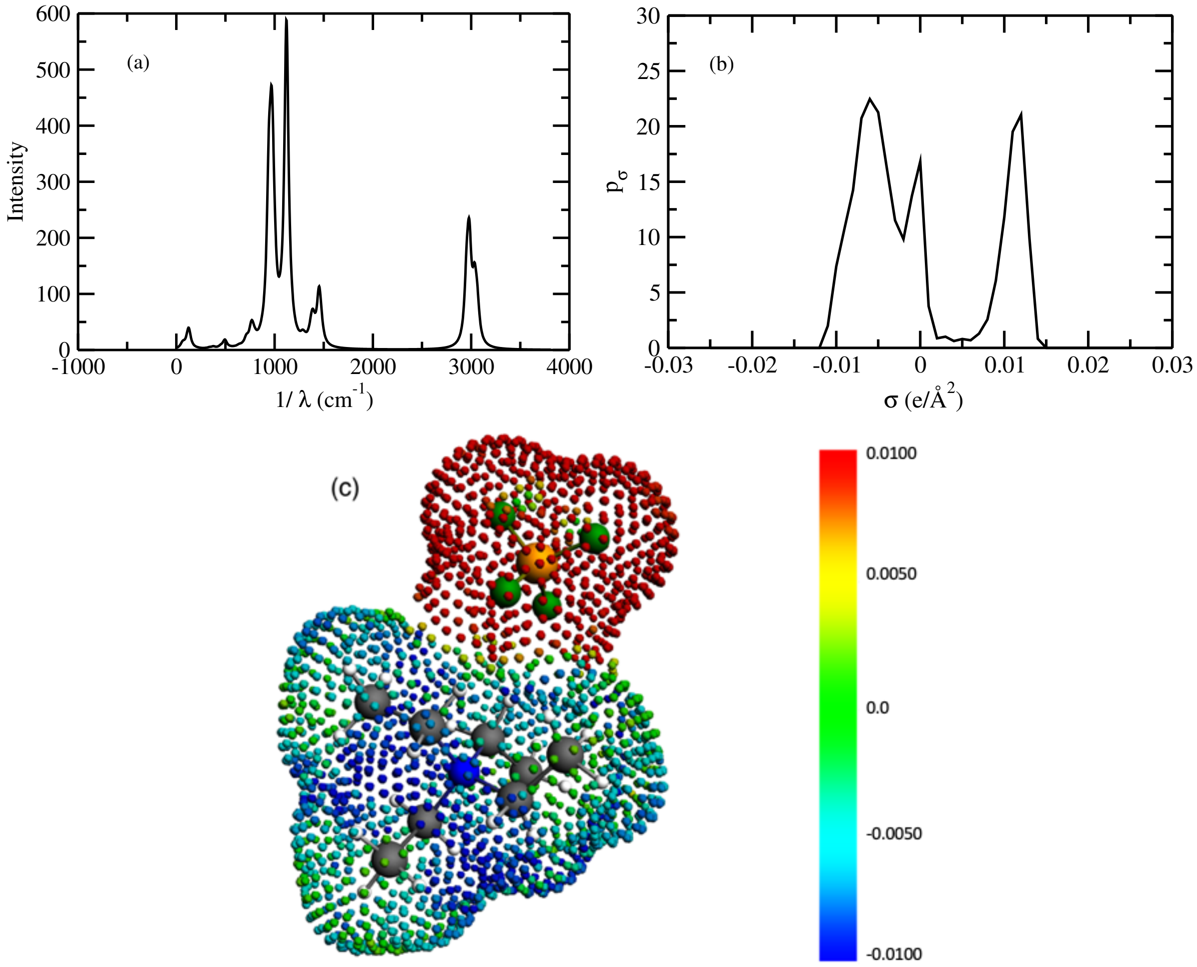

Figure. S 4: In figs.(a) analytical frequency, (b) sigmaprofile and (c) COSMO points of [Tea][BF4] ionic liquid.

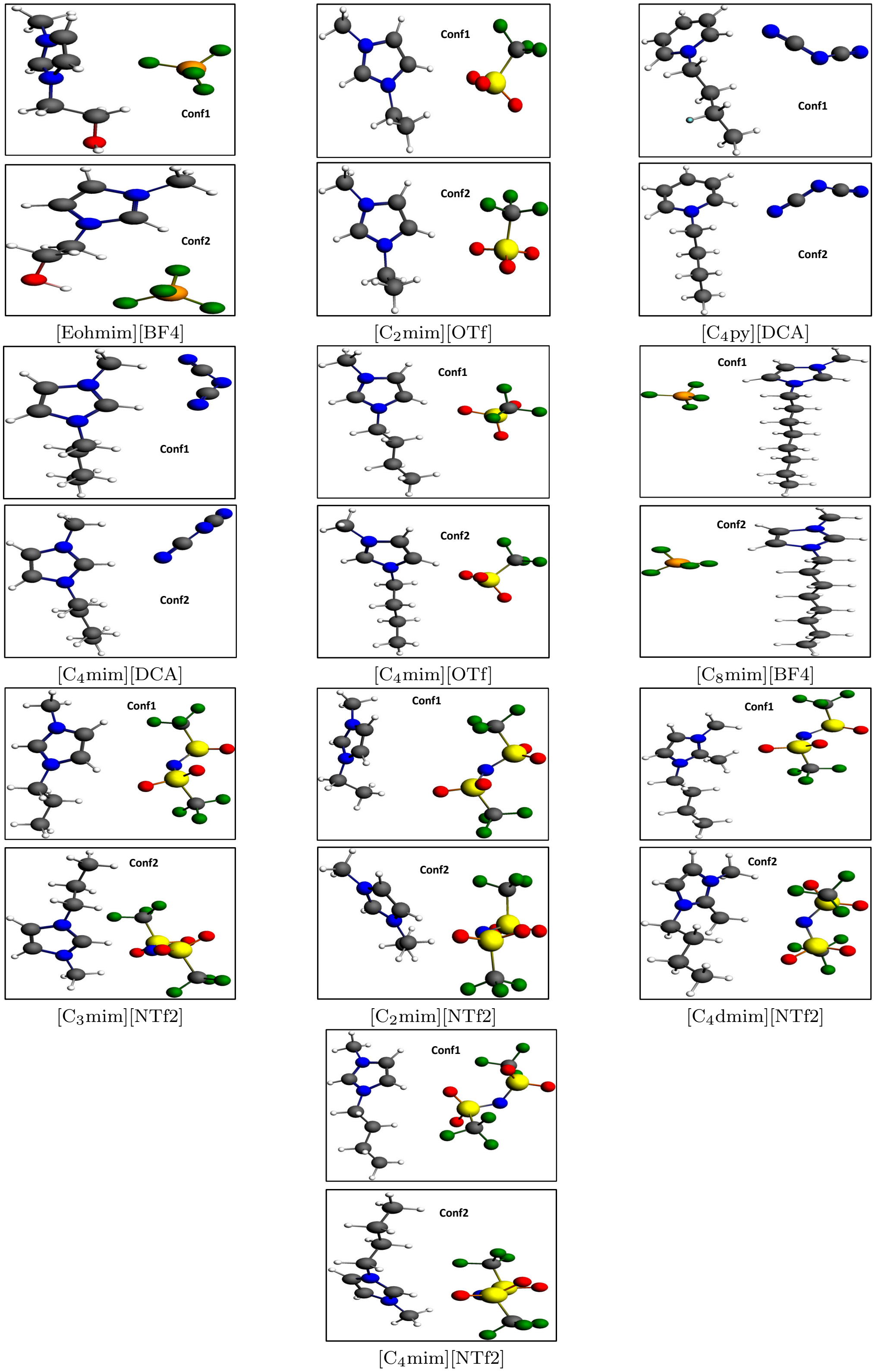

Figure. S 5: Two different conformations of 10 ionic liquids.

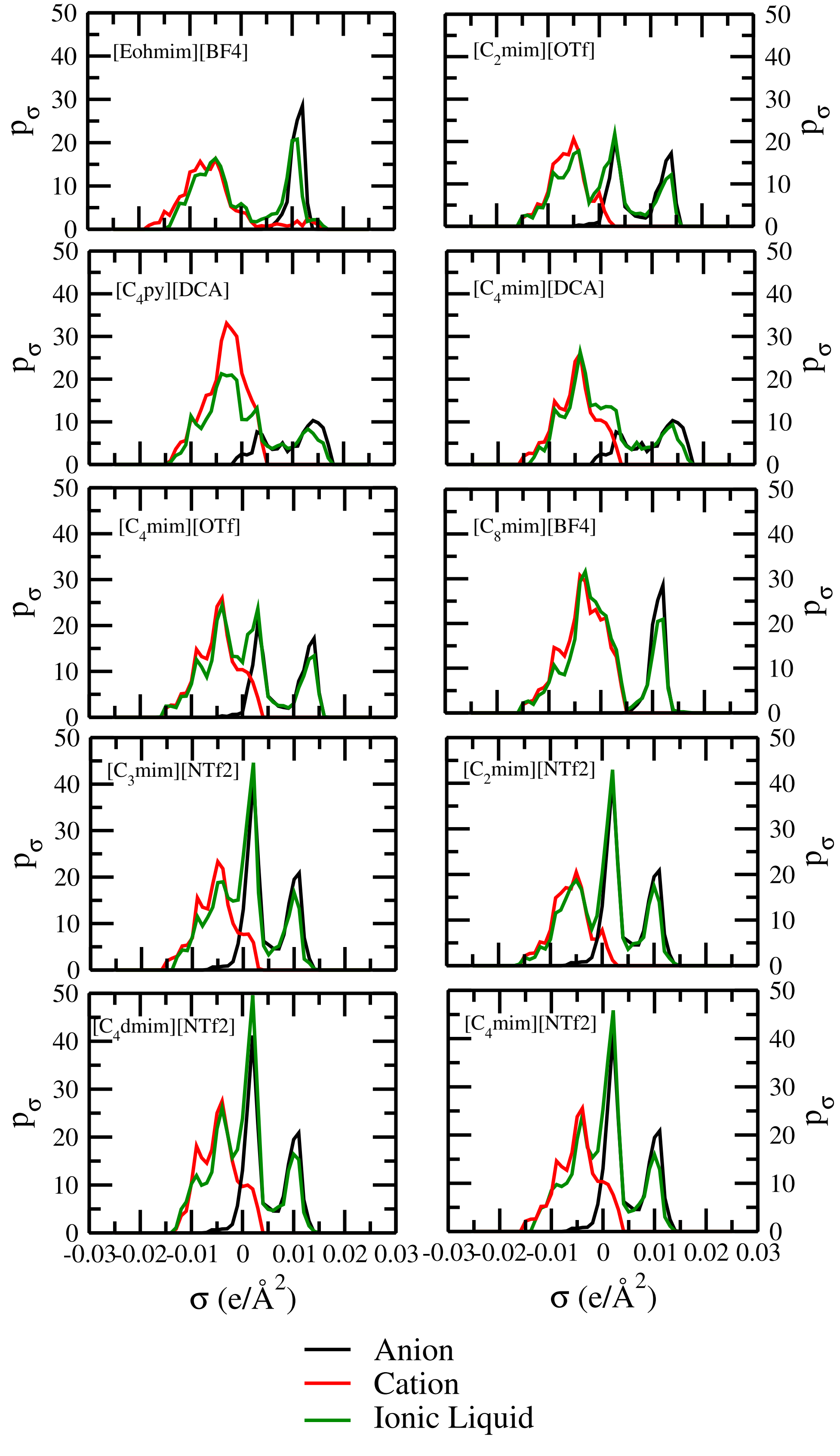

Figure. S 6: Sigmaprofile of ionic liquids for separate cation, anion and total ionic liquid.

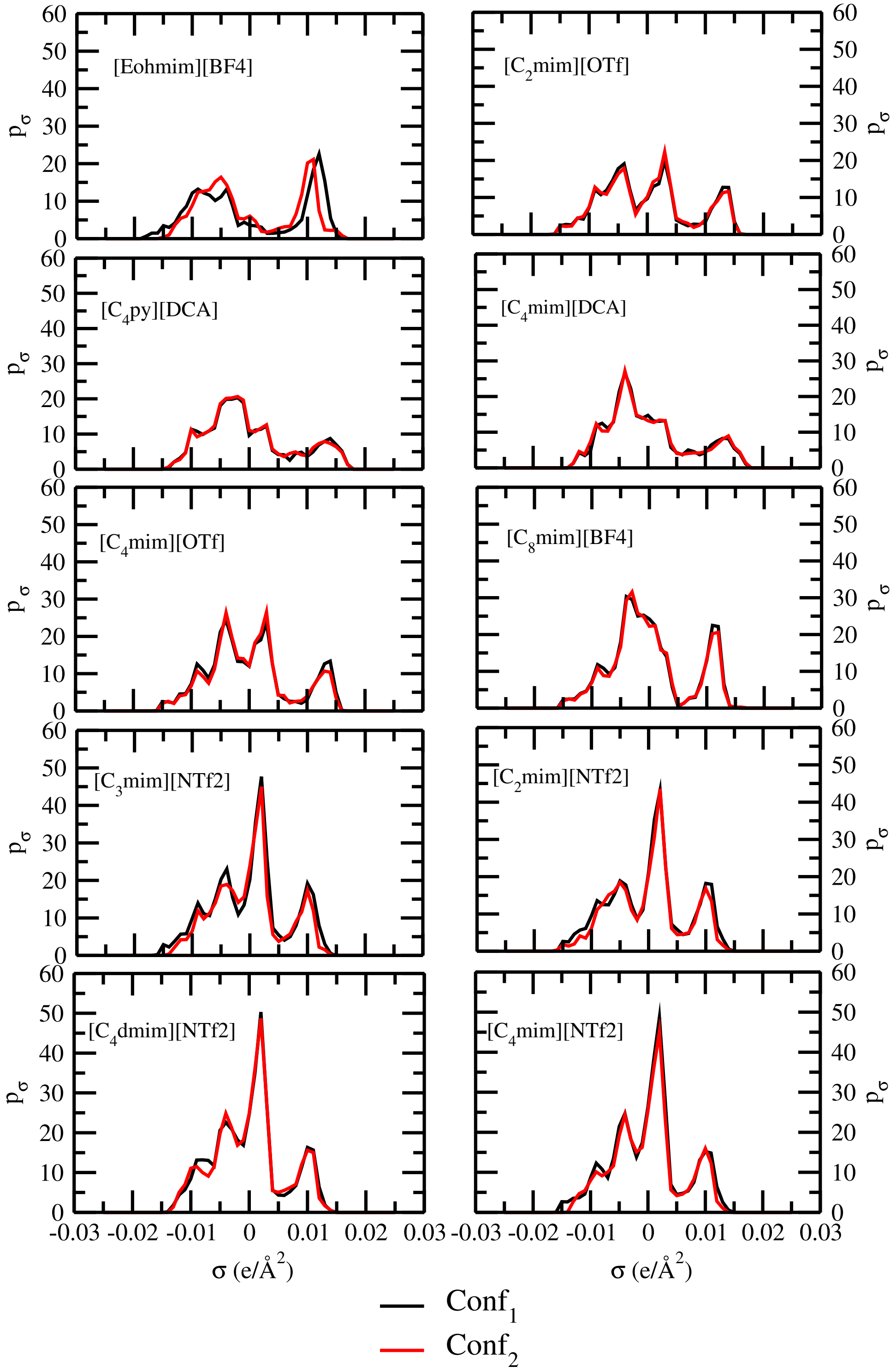

Figure. S 7: Sigmaprofile of two different conformations of ionic liquids

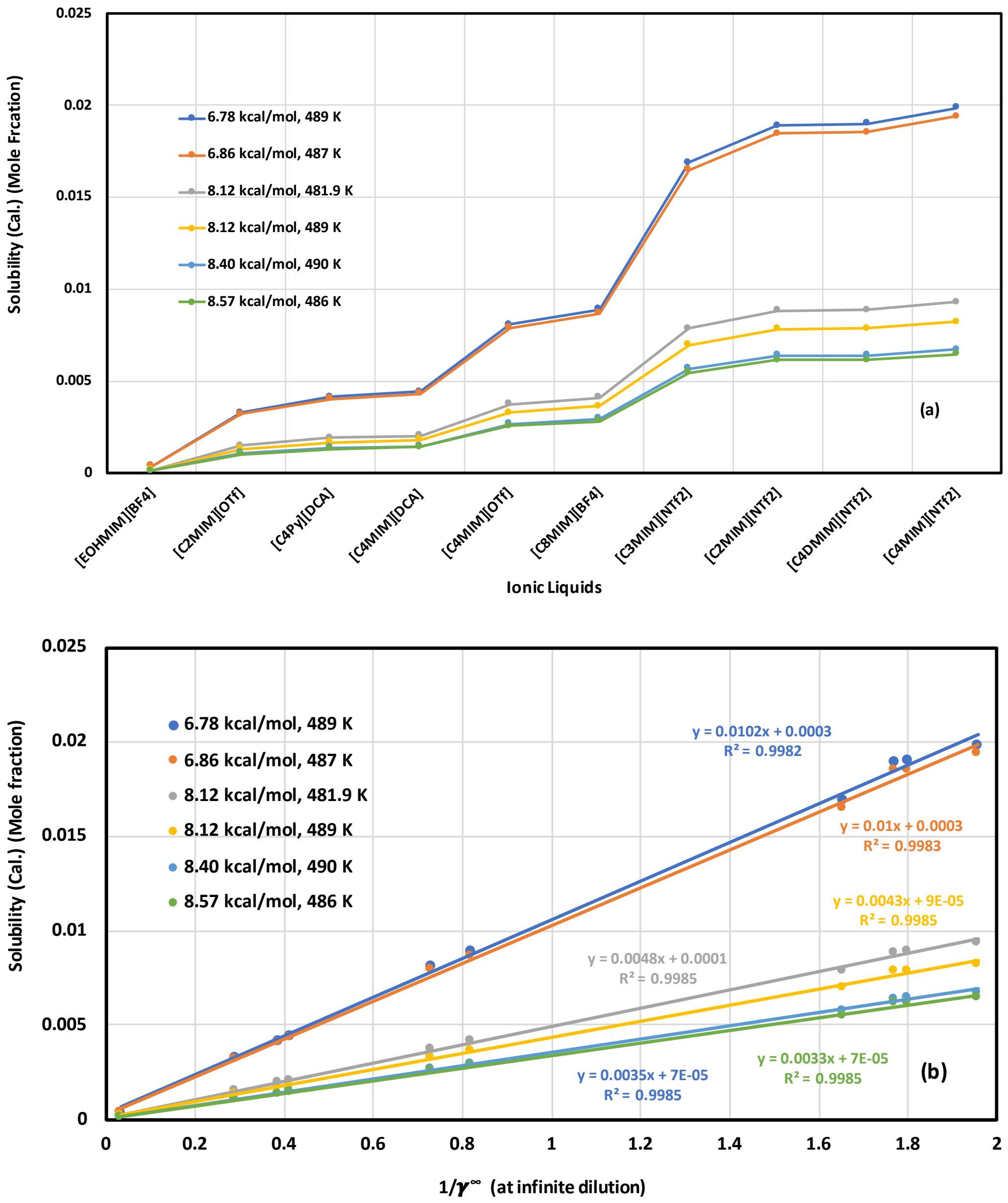

Figure. S 8: In Fig.(a) solubility of $Cr(acac)_3$ in 10 ionic liquids and in fig.(b) the solubility of $Cr(acac)_3$ in 10 ionic liquids is correlated with the activity coefficient at infinite dilution of the metal complex in ionic liquids for different heat of fusions and melting temperatures.
